# *In vitro* investigation of the host-pathogen interaction in human strongyloidiasis at the intestinal level

**DOI:** 10.3389/fcimb.2026.1981291

**Published:** 2026-10-28

**Authors:** Michela Deiana, Nicole Padovani, Elisabetta Vezzelli, Marcello Manfredi, Elena Locatelli, Lucas Moron Dalla Tor, Niccolò Ronzoni, Tamara Ursini, Chiara Piubelli, Dora Buonfrate, Natalia Tiberti

**Affiliations:** 1 Department of Infectious, Tropical Diseases and Microbiology, IRCCS Sacro Cuore Don Calabria Hospital, Negrar di Valpolicella, Italy; 2 Department of Engineering for Innovation Medicine, University of Verona, Verona, Italy; 3 Department of Translational Medicine, University of Piemonte Orientale, Novara, Italy; 4 Institute for Molecular and Translational Cardiology (IMTC), IRCCS Policlinico San Donato, Milano, Italy; 5 Department of Medicine and Surgery (DiMeC) - Integrated Biotechnology complex, University of Parma, Parma, Italy

**Keywords:** Caco-2 cells, extracellular vesicles, host-pathogen interaction, proteomics, *Strongyloides stercoralis*, transcriptomics

## Abstract

**Introduction:**

Human strongyloidiasis is a chronic long-lasting infection caused by the helminth parasite *Strongyloides stercoralis*, characterised by a peculiar autoinfective cycle. The interplay between the parasite and the host should hence entail mechanisms of adaptation to tolerate the chronic presence of this nematode. However, the current knowledge of the mechanisms underpinning this interaction remains limited. To gain novel insights into the interaction between *S. stercoralis* and the human host, we employed multi-omics approaches to explore the modulation induced by *S. stercoralis* infective larvae (iL3) and their extracellular vesicles (EVs) on human intestinal epithelial cells.

**Methods:**

*S. stercoralis* iL3 isolated from a clinical faecal sample were maintained *in vitro* for up to one week to allow the release of EVs in the conditioned medium. Human intestinal epithelial cells (Caco-2) were then exposed to either iL3 or their EVs for 6h, 24h or 72h.

**Results:**

In our settings, different responses are triggered by the two iL3 or EVs over time. After 6 hours of coincubation, iL3 induced the release of pro-inflammatory cytokines, namely IL-8and CXCL10, while EVs only induced the release of CXCL10, with lower intensity compared to live larvae. Evaluation of gene expression modulation by transcriptomics and proteomics revealed that live larvae induced cellular activation and a pro-inflammatory like profile that peaked 72h post-exposure, while EVs displayed an earlier effect with modulation in mRNA and protein expression mostly 24h post-treatment and leading to a predominant inhibition of cell activation, metabolism and immune response.

**Discussion:**

These findings highlight distinct effects mediated by iL3 and EVs in the local host-pathogen interaction during human strongyloidiasis. By showing a primary down-modulation of cell activation, we hypothesise that EVs may play a role in immune evasion. More in-depth investigations are needed to validate these findings and further elucidate the role of parasite-derived EVs in the pathophysiology of human strongyloidiasis.

## 1 Introduction

*Strongyloides stercoralis* is a nematode parasite responsible for human strongyloidiasis, a neglected tropical disease affecting disadvantaged populations living in areas of inadequate human waste disposal (Gordon et al., 2024). Due to the limited available data from endemic areas, the actual prevalence of strongyloidiasis is unknown (Gordon et al., 2024); however, the latest estimates indicate that 380–600 million people might be infected worldwide (Buonfrate et al., 2020; Fleitas et al., 2020).

*S. stercoralis* has a complex life cycle encompassing both free living and parasitic stages (Castelletto et al., 2024). Infection is acquired through direct contact with soil contaminated with free-living infective larvae (iL3). Following skin penetration, iL3 migrate through the lungs, ascend the respiratory tree and are swallowed. They then reach the small intestine, where they establish as parthenogenic adult female worms. In the mucosa, females release embryonated eggs, which hatch releasing rhabditoid larvae (L1) with faeces.

Due to a peculiar auto-infective cycle, *S. stercoralis* causes a long-lasting chronic infection, which can present with subtle, unrelenting symptoms of different grade of severity (Barroso et al., 2019; Buonfrate et al., 2019; Gordon et al., 2024). The infection may also go unnoticed due to very mild, intermittent symptoms. However, alterations of the host immune status - such as immunosuppression – can lead to severe strongyloidiasis (dissemination or hyper infection), which is life-threatening (Gordon et al., 2024). This pathophysiological feature highlights the importance of the cross-talk between the host immune system and the parasites for disease control, the mechanisms of which are however yet to be fully deciphered.

Helminth parasites have evolved intricate mechanisms of interaction with their host to manipulate the immune response and survive relatively adverse conditions, without creating excessive damage to the host (Maizels and Yazdanbakhsh, 2003). The intestinal epithelium plays a central role in the response to helminth infections, being an important regulator of the type 2 immune response (Inclan-Rico and Siracusa, 2018). Nonetheless, how these multicellular parasites shape this barrier tissue remains poorly explored (Karo-Atar et al., 2023). Most of the evidence regarding the intestinal response to *Strongyloides* spp. has been collected through studies using laboratory strains and animal models. In fact, studies in rodents using *S. ratti* or *S. venezuelensis* infections have been essential to reveal key aspects of *Strongyloides* spp. infections, including the central role of type 2 innate lymphoid cells (ILC2) in the anti-helminthic protective immunity and that different immune mediators are required for infection control at the different sites during tissue migration (Yasuda et al., 2014; Bouchery et al., 2019; Meiners et al., 2020; Breloer and Linnemann, 2024; Heepmann et al., 2026). However, although *S. ratti* rodent infection is undeniably useful to decipher the host-parasite crosstalk, it can only partly recapitulate the features of the human infection. For instance, *S. ratti* does not undergo the auto-infective cycle and therefore infection results in parasite clearance mediated by the type 2 immune response (Breloer and Linnemann, 2024).

Helminth-derived excretory/secretory products (ESP) are also important mediators of infection control, and have been supposed to contribute to the establishment of chronic infection without creating excessive damage to the host (Allen and Wynn, 2011; Maizels et al., 2018). Although not extensively investigated in strongyloidiasis, there is evidence showing that *S. ratti-*derived ESP carry stage-specific proteins (Soblik et al., 2011) and interfere with cytokine secretion by ILC2 (Hartmann et al., 2026).

An important component of helminth-derived ESP is represented by extracellular vesicles (EVs) (Drurey and Maizels, 2021). These submicron elements are rapidly emerging as important mediators of the host-pathogen interaction (Sotillo et al., 2020; Yeshi et al., 2022; White et al., 2023) due to their ability to modulate the host response (Buck et al., 2014; Coakley et al., 2017; Duque-Correa et al., 2020) and to induce partial protection in vaccination models (Trelis et al., 2016). Recently, we demonstrated that, when maintained *in vitro*, *S. stercoralis* iL3 release EVs carrying a cargo of miRNAs and other smallRNAs (Deiana et al., 2026). Building upon this knowledge, here we explored the potential functional effects mediated by *S. stercoralis* iL3 and their derived EVs on a human cell line from colorectal adenocarcinoma (Caco-2) using multi-omics approaches, with the overarching goal of shedding new light on the modulation induced by *S. stercoralis* on human intestinal epithelial cells.

## 2 Materials and methods

### 2.1 Parasite isolation, maintenance and genotyping

*S. stercoralis* iL3 were isolated from fresh stools collected for diagnostic purposes from an infected patient after 6 days of agar plate culture (Buonfrate et al., 2022) (**Supplementary Figure S1**). Only parasites derived from diagnostic leftovers were used. Larvae were axenised with 100 U/ml penicillin, 100 µg/ml streptomycin and 0.625 µg/ml amphotericin B (all from Gibco, Thermo Fisher Scientific, Waltham, MA, USA) for 2 h at 4 °C, and then maintained in 1 μm trans-well inserts in *S. stercoralis* medium (DMEM supplemented with 5% heat-inactivated and exosome-depleted Fetal Bovine Serum (ED-FBS), 100 U/ml penicillin, 100 µg/ml streptomycin, 0.625 µg/ml amphotericin B, 2 mM L-Glutamine, 1x MEM Non-Essential Amino Acids Solution, 1 mM HEPES Buffer - all from Gibco, Thermo Fisher Scientific). Larval density was approximately 1, 000 larvae/ml. Conditioned medium was collected daily from the lower compartment, centrifuged 30 min at 2, 000 x*g* to remove debris and stored at -80 °C until further use.

The genotype of the clinical isolate was assessed as reported by Barratt et al (Barratt et al., 2021) and assigned to the H1_A(i)_VI genotype. Sequences have been uploaded to GenBank with the accession numbers PZ360109 (COX1), PZ327591 (HVR-I) and PZ327586 (HVR-IV).

### 2.2 Extracellular vesicle enrichment and characterisation

iL3-EVs were enriched by differential ultracentrifugation (UC) from 26 ml of conditioned medium, as already reported (Deiana et al., 2026). Briefly, conditioned medium was centrifuged at 10,000 x*g* for 10 minutes and then ultracentrifuged at 100,000 x*g* for 2 hours at +4 °C using a Fiberlite F50L-24 x 1.5 Fixed-Angle Rotor on a SorvallTM WX ultracentrifuge (Thermo Scientific). EV pellet was washed with 0.22 μm-filtered PBS at 100,000 x*g* for 2 hours at +4 °C. Washed pellet was re-suspended in PBS and filtered through 0.22 μm spin filters (Corning). Protein concentration was measured by Qubit protein assay (ThermoFisher Scientific).

Particle size and concentration were analysed by Nanoparticle Tracking Analysis (NTA) using a NanoSight NS300 instrument (Malvern Panalytical, Malvern, UK) equipped with a 488 nm laser and a scientific CMOS camera. EVs were diluted 1:200 to obtain a concentration and particles/frame within the recommended measurement range. Three consecutive videos of 60s per sample were acquired with a syringe pump speed set to 20 and subsequently analysed using the NTA 3.4 software (Malvern).

To visualise EVs by transmission electron microscopy (TEM), one drop of sample solution (approximately 25 µl) was placed on a 400-mesh holey film grid and stained with 2% uranyl acetate for 2 minutes. The sample was observed with a Tecnai G2 (FEI) transmission electron microscope operating at 120 kV. Images were captured with a Veleta (Olympus Soft Imaging System) digital camera.

### 2.3 Caco-2 cell maintenance and stimulation

Human colon adenocarcinoma cell line (Caco-2, HTB-37™, ATCC, Manassas, VA, USA) was maintained in culture at 37 °C in 5% CO_2_ as described elsewhere (Natoli et al., 2012). Cells were negative for *Mycoplasma* contamination.

Cells were seeded in 24 well plates at a density of 200,000 cells/well, and maintained for 3 days to reach confluence before exposure to the different stimuli for 6h, 24h or 72h.

Cells were exposed to the following stimuli: iL3 (mean: 14.25 larvae/well; range: 7–30 larvae/well, **Supplementary Table S1**); iL3-EVs (2.4x10^8^ EVs/well corresponding to 1.1 μg of proteins/well); 20 ng/mL IFNγ (Gibco), used as a pro-inflammatory control (Shi et al., 2018). Untreated cells incubated with medium only were used as resting condition. Larvae maintained without cells were used as controls for cytokine release to rule out potential cross-reactivity or non-specific signals derived from larval secretions. Each stimulus (or resting) was tested in four replicates; larvae without cells were tested in duplicate. Cell viability was assessed with the CyQUANT™ XTT Cell Viability Assay (Invitrogen), while larval viability was evaluated by microscopic examination of larval motility at each time point. The study design is reported in **Figure 1**.

**Figure 1:**
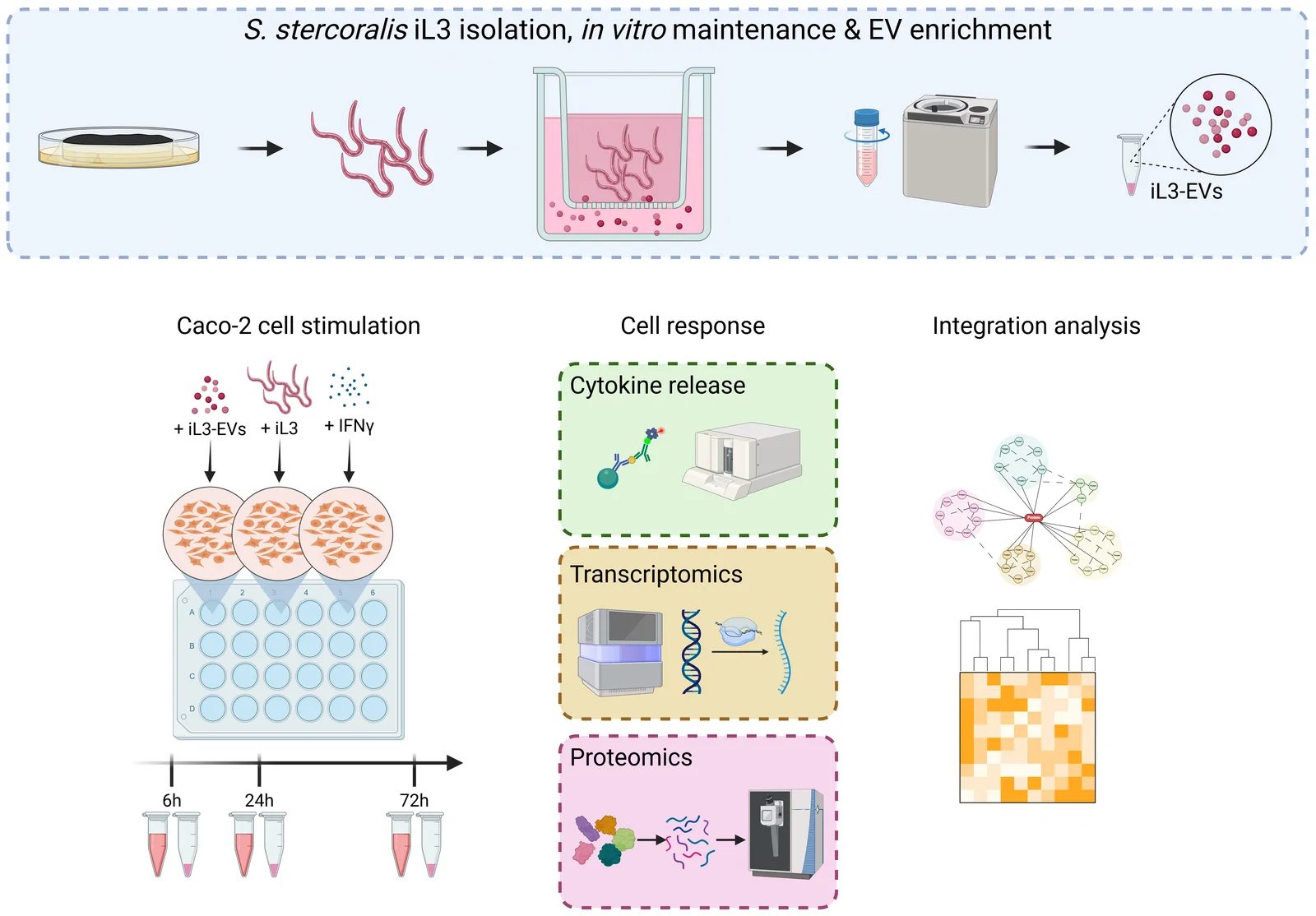
Study design. The workflow for *S. stercoralis* iL3 isolation from a clinical sample, *in vitro* maintenance and enrichment of EVs released in the conditioned medium is reported in the upper part of the figure. To evaluate the host-pathogen interaction, Caco-2 cells were exposed to EVs, live iL3 or IFNγ (positive inflammatory control) for 6h, 24h and 72h. Downstream analyses included measurement of cytokine release in the conditioned medium, transcriptomics and proteomics of Caco-2 cells. Created in https://BioRender.com.

After stimulation, conditioned medium was collected from each well and centrifuge at 500 x*g* for 5 min to remove cell debris and stored at -80 °C until further use. Cells for transcriptomics were washed twice in PBS and harvested by gentle scraping in cold PBS prior to storage at -80 °C. Cells for proteomics were washed twice in PBS, incubated for 5 min in PhosphoSafe Extraction Reagent (Novagen^®^) and centrifuged for 5 min at 16,000 x*g* at 4 °C. Clear supernatants containing extracted proteins were stored at -80 °C.

### 2.4 Cytokine measurement

The concentration of CXCL10, IL-6, IL-8, IL-25, IL-33 and thymic stromal lymphopoietin (TSLP) were determined in the conditioned medium using a custom Luminex^®^ Discovery assay (R&D Systems) on a Luminex^®^ 200 analyser and analysed using the Luminex xPONENT^®^ software v4.3. Four technical replicates were assessed for each condition, except for larvae without cells for which two technical replicates were performed. To avoid missing values, samples having measured concentration below or above the assay limit of detection were assigned arbitrary values as reported elsewhere (Tiberti et al., 2026). Cytokines having more than 90% of values below the limit of detection were excluded from further analysis.

### 2.5 Transcriptomics

Caco-2 cells exposed to EVs, iL3, or IFNγ, along with untreated resting cells, were analysed by transcriptomic profiling. Total RNA was extracted from two technical replicates for each experimental condition using the miRNeasy Mini Kit (Qiagen) according to manufacturer’s instructions. RNA concentration was determined using the Qubit™ 4 Fluorometer (Qubit RNA HS Assay Kit, ThermoFisher Scientific), whereas RNA integrity was evaluated using the 4200 TapeStation System with the High Sensitivity D1000 screen tape (Agilent). All samples had RNA Integrity Number (RIN) > 6.

RNA-seq libraries were generated from 100 ng of total RNA using the Illumina Stranded Total RNA with Ribo-Zero Plus Library Prep Kit according to the manufacturer’s protocol (Illumina). Library concentration and quality were evaluated using the Qubit RNA HS Assay and the 4200 TapeStation System using the High Sensitivity D1000 ScreenTape. Libraries were sequenced on an Illumina NextSeq1000 platform (Illumina), using a paired-end 76-bp strategy (PE76).

Raw reads were quality-checked by FastQC (v.0.12.1). Adapter sequences and low-quality bases were removed prior to downstream analyses. Transcript-level quantification was performed with Salmon (v1.10.1) using the human reference genome GRCh38 (parameters -l A). Gene counts were generated using the GENCODE v46 gene annotation file. Library size differences were normalized using the Trimmed Mean of M-values (TMM) normalization method using the edgeR package (v.4.4.2). Differential genes expression analysis was performed using limma packages (v. 3.62.2) in R (v. 4.4.1).

### 2.6 Proteomics

Caco-2 cells exposed to EVs, iL3, or IFNγ, along with untreated resting cells, were analysed by quantitative proteomics. Proteins obtained from two technical replicates for each condition were used for proteomics analyses. Protein concentration was determined using the bicinchoninic acid (BCA) assay, and 10 µg of proteins from each sample were processed. Protein reduction with 5 µL of dithiothreitol (DTT; 5 mg/mL) at 65 °C for 15 min. Cysteine residues were subsequently alkylated with 50 µL of iodoacetamide (IAA; 15 mg/mL) for 30 min at room temperature in the dark. Samples were then diluted with 200 µL of urea buffer containing 8 M urea and 100 mM Tris-HCl (pH 8.0) to establish denaturing conditions before loading onto the centrifugal filters. Centrifugal filters with a 10-kDa molecular-weight cut-off were conditioned with 100 µL of 60% methanol and centrifuged at 14,000 x*g* for 10 min. The diluted samples were loaded onto the filters and centrifuged at 14,000 x*g* for 25 min. The filters were subsequently washed three times with 200 µL of 8 M urea, in 100 mM Tris-HCl (pH 8.0), with centrifugation at 14,000 x*g* for 25 min after each wash. Buffer exchange was performed by washing the filters twice with 200 µL of 50 mM ammonium bicarbonate under the same centrifugation conditions. The collection tubes were then replaced, and 100 µL of 50 mM ammonium bicarbonate was added to each filter. Proteins were digested overnight at 37 °C with sequencing-grade trypsin at an enzyme-to-protein ratio of 1:80. Peptides were recovered by adding 50 µL of 50 mM ammonium bicarbonate and centrifuging the filters at 14,000 x*g* for 15 min. This elution step was repeated, and the resulting filtrates were combined. The peptide mixtures were acidified with formic acid to a final concentration of 1% (v/v), corresponding to a pH below 3, desalted by solid-phase extraction (Discovery^®^ DSC-18 solid phase extraction 96-well plate, 25 mg/well, Sigma-Aldrich Inc., St.Louis, MO, USA). Peptides were eluted with 80% acetonitrile containing 0.1% formic acid, dried by vacuum, and reconstituted in mobile phase A, consisting of 0.1% formic acid in water, before LC–MS/MS analysis.

One µl of the sample was injected into the nano column and analysed on an Ultimate 3000 RSLC nano coupled directly to an Orbitrap Exploris 480 with a High-Field Asymmetric Waveform Ion Mobility Spectrometry System (FAIMS pro) (all Thermo Fisher Scientific). In particular, peptides were eluted from the column with the following gradient: the elution ramp ranged from 6% to 95% mobile phase B (80% acetonitrile, 0.1% formic acid) over 41 min with a flow rate of 500 nL/min, followed by an equilibration with 6% mobile phase B for 1 min. The acquisition of each sample lasted 41 minutes and was performed in data-independent modality. The total recording of the MS spectra was carried out only in positive resolution with a high voltage of 2500 V and the FAIMS interface in standard resolution, with a CV of -45V. The isolation window was set to 8 m/z and the window overlap to 1 m/z; precursor mass range was between 400 and 900, orbitrap resolution 30000 and RF Lens at 50% and HCD collision energy of 27%. Finally, the normalised AGC target was set to 1000, the maximum injection time was 25 ms, and microscan was 1.

For the data processing of the DIA-acquired raw data, DIA-NN (version 1.8.1) was exploited: identification was performed using a spectral library built from a library-free search of the entire dataset against Human UniProt protein database (downloaded on February 10^th^, 2026). In addition, “deep learning-based spectra, RTs and IMs prediction” were enabled. Enzyme was set to Trypsin/P, precursors of charge state 1–4, peptide lengths 7–30 and precursor m/z 400–900 were considered with maximum two missed cleavages. Carbamidomethylation on C was set as fixed modification and Oxidation on M as variable modification, using a maximum of two variable modifications per peptide. FDR was set to 1% and match between run was also enabled. The mass spectrometry proteomics data have been deposited to the ProteomeXchange Consortium via the PRIDE (Perez-Riverol et al., 2019) partner repository with the dataset identifier PXD082958.

### 2.7 Differential expression and pathway analyses

For both transcriptomic and proteomic datasets, expression values were obtained from the average of the two technical replicates. Stimulated conditions were then compared with untreated resting cells using paired t-test. Features showing a p-value <0.05 and an absolute fold-change (FC) ≥2 were considered differentially expressed and visualised through Volcano plots (**Supplementary Figures S2, S3**). Principal Component Analyses (PCA) and hierarchical clustering analyses (HCA) were performed using the FC of each stimulus to the resting condition. Statistical analyses were performed with R studio v4.4.1.

Pathway analyses were performed using Ingenuity Pathway Analysis (IPA) (Qiagen) (Kramer et al., 2014) on mRNA and proteins identified in cells exposed to EVs or iL3. In order to allow an appropriate number of entries, core expression analysis was performed considering the following filtering criteria: *i)* paired p-value <0.05 and |FC| ≥2 for mRNA; *ii)* |FC| ≥2 for proteins. Pathways showing an enrichment p-value <0.05 and a |z-score| ≥2 (indicative of the confidence of pathway modulation) were considered as significantly represented among the dataset. All pathways significantly represented in at least one time point for each stimulus (i.e., iL3 or EVs) were further considered.

## 3 Results

### 3.1 EVs can be efficiently enriched from iL3-conditioned medium

The presence of EVs in the conditioned medium was evaluated by NTA following enrichment by UC (**Figures 2A, B**). A population of particles with mean size of 132.1 nm and mode size 106.0 nm was observed. Negative staining and TEM imaging confirmed the presence of cup-shaped particles with a size range consistent with NTA measurements (**Figure 2C**).

**Figure 2:**
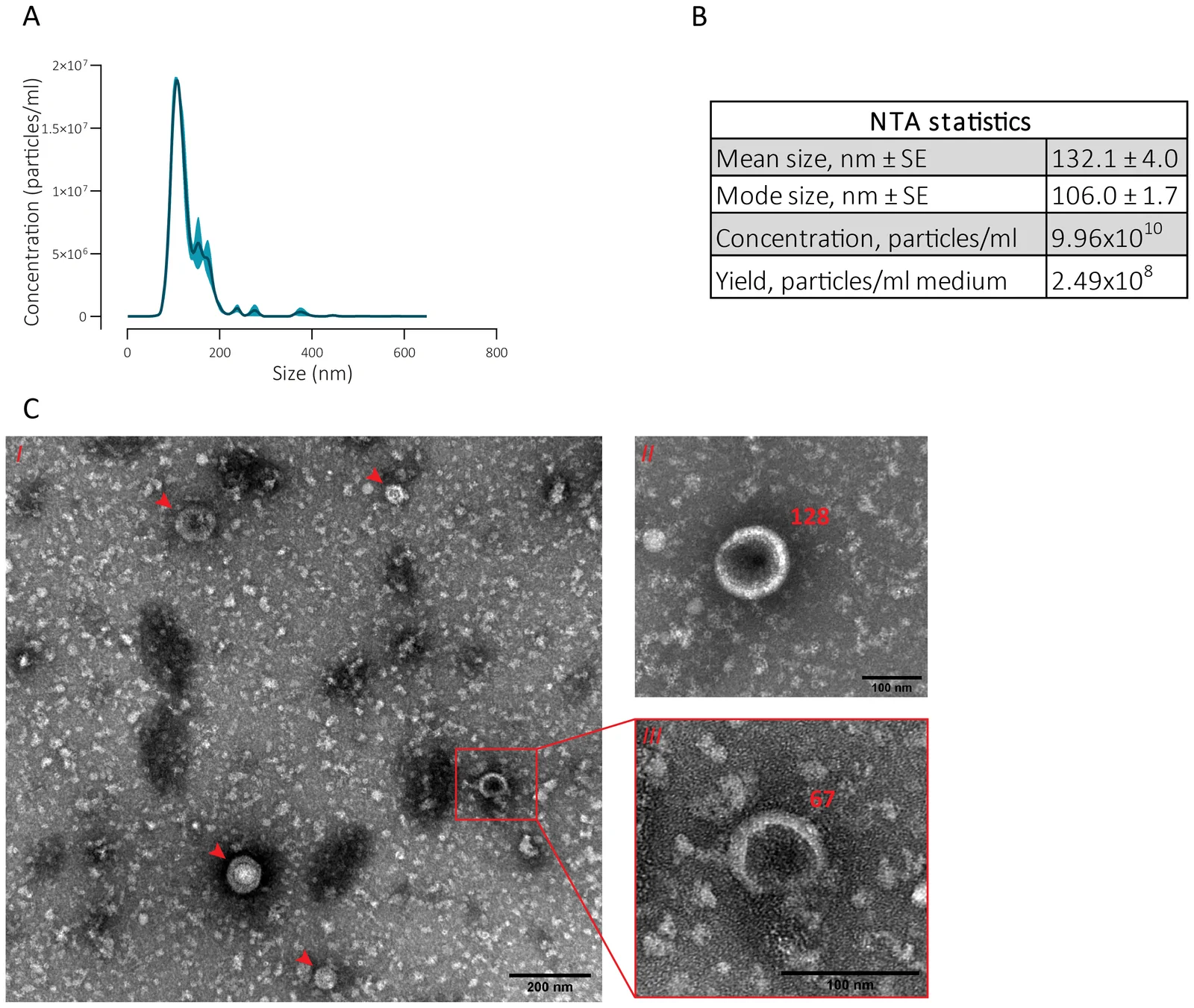
Morphological evaluation of EVs released by *S. stercoralis* iL3 following *in vitro* maintenance. **(A, B)** NTA measurement of size distribution and particle concentration. **(C)** EV imaging by transmission electron microscopy. Scale bars: 200nm in I, 100nm in II and III. Red arrowheads indicate EVs, numbers on the panels represent vesicle diameters in nm.

### 3.2 Caco-2 cells release pro-inflammatory cytokines in response to *S. stercoralis* iL3 and their EVs

To exclude potential cytotoxic effects induced by live larvae or parasite-derived EVs, Caco-2 cell viability was evaluated by XTT assay. No significant reduction in cell viability was detected in cells exposed to either iL3 or EVs; a modest reduction was observed in cells exposed to iL3 at 72h even though overall viability remained high and not statistically different from resting untreated cells (**Supplementary Figure S4**).

Among the assessed cytokines, IL-6, IL-33 and TSLP were not released by Caco-2 cells in any of the tested conditions, while IL-25 was detected at very low concentrations only in IFNγ-treated samples (**Supplementary Table S2**). CXCL10 and IL-8 were instead detected in Caco-2 conditioned medium (**Figure 3**). Both cytokines were negative when measured in the conditioned medium of iL3 maintained without cells (**Figure 3A**). Compared to unstimulated cells, both iL3 and EVs induced the release of significantly higher amount of CXCL10 by Caco-2 cells at both 24h and 72h (p<0.01, Mann-Whitney U test), even though an overall lower CXCL10 abundance was observed in cells exposed to EVs compared to iL3 (**Figure 3B**). IL-8 was released in the conditioned medium only in response to iL3 and its concentration in iL3-treated cells compared to resting cells was significantly higher at both 24h and 72h (p<0.01, Mann-Whitney U test) (**Figure 3C**).

**Figure 3:**
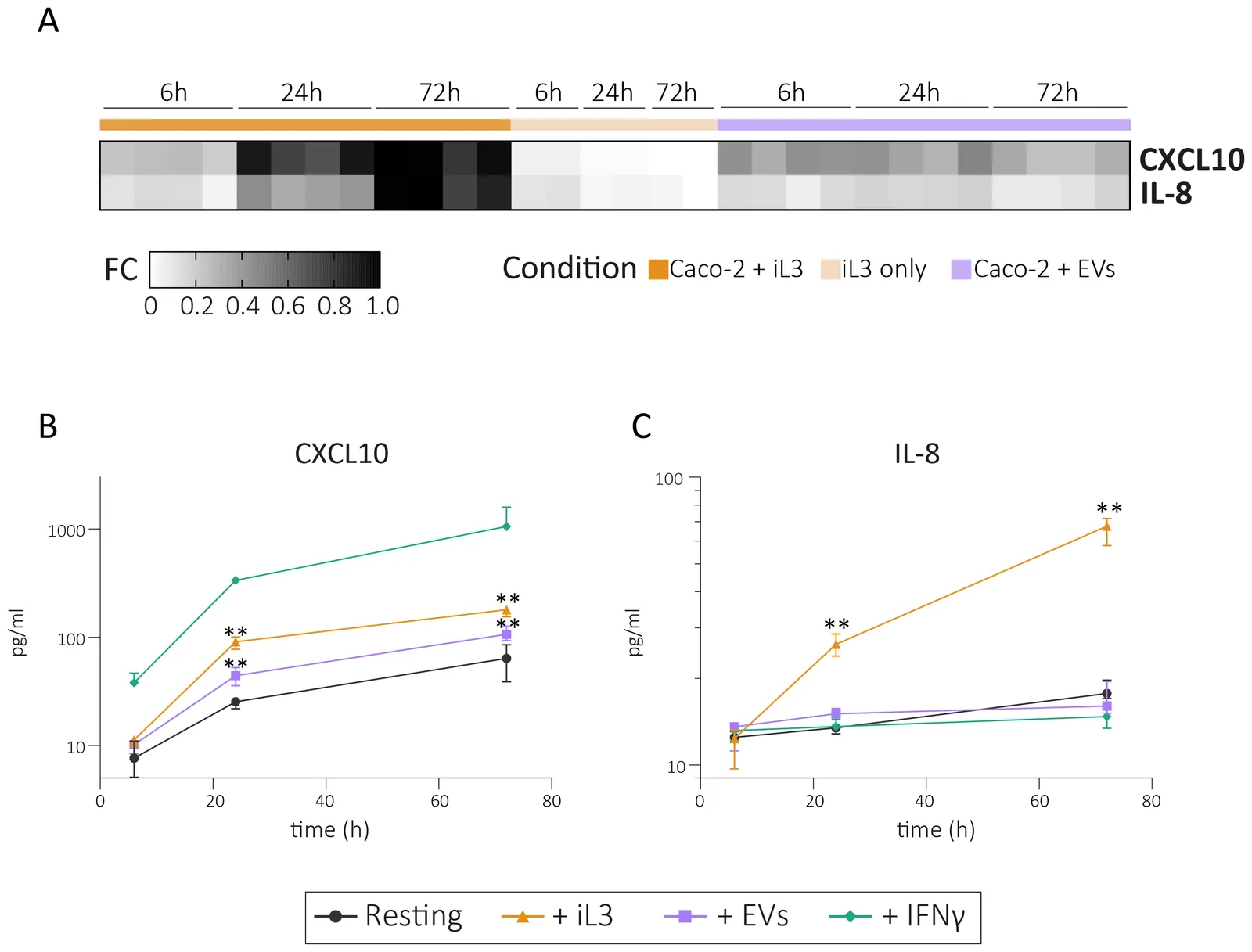
Cytokines released by Caco-2 cells in the conditioned medium. **(A)** Heat map showing the levels of CXCL10 and IL-8 measured in the conditioned medium from Caco-2 cells exposed either to iL3 or EVs. Conditioned medium of larvae maintained without cells (i.e., iL3 only) was also assessed. Data are reported as FC (0–1 min-max scaled) of the resting condition. For each condition, values obtained for each technical replicate are reported. **(B, C)** Detailed kinetics of CXCL10 and IL-8 measured in the medium of Caco-2 cells exposed to the different stimuli (i.e., iL3, EVs or IFNγ). Values measured in untreated cells (i.e., resting) are also reported. Symbols and error bars indicate the median ± interquartile range (IQR). Stars on the graphs represent the statistical significance for the comparison of iL3 or EVs relative to resting cells (**Mann-Whitney U test p-value <0.01).

### 3.3 Transcriptomic profiles of Caco-2 cells are modulated following exposure to *S. stercoralis* iL3 or EVs

To investigate the modulation in the transcriptional response induced by *S. stercoralis* iL3 or parasite-derived EVs, RNA-seq analysis was performed at 6, 24 and 72h post-stimulation. IFNγ-treated cells were included as a positive control to monitor the induction of an inflammatory response. RNA-seq analysis identified a total of 20, 156 transcripts (**Supplementary_Table 1_Transcriptomics**).

PCA revealed a temporal and stimulus-dependent transcriptional response (**Figure 4A**). The first two principal components accounted for 36.6% and 20.4% of the total variance, respectively. No clear separation between treatments or time points was observed; however, the three samples exposed to EVs clustered together, while cells stimulated with iL3 or IFNγ collected at 24h clustered closely together regardless of treatment. In contrast, samples collected at 6h showed the greatest separation, particularly the IFNγ-treated cells, which displayed the strongest transcriptional shift. At 72h, EV-, iL3-, and IFNγ-treated samples clustered more closely, indicating a partial convergence of transcriptional responses over time.

**Figure 4:**
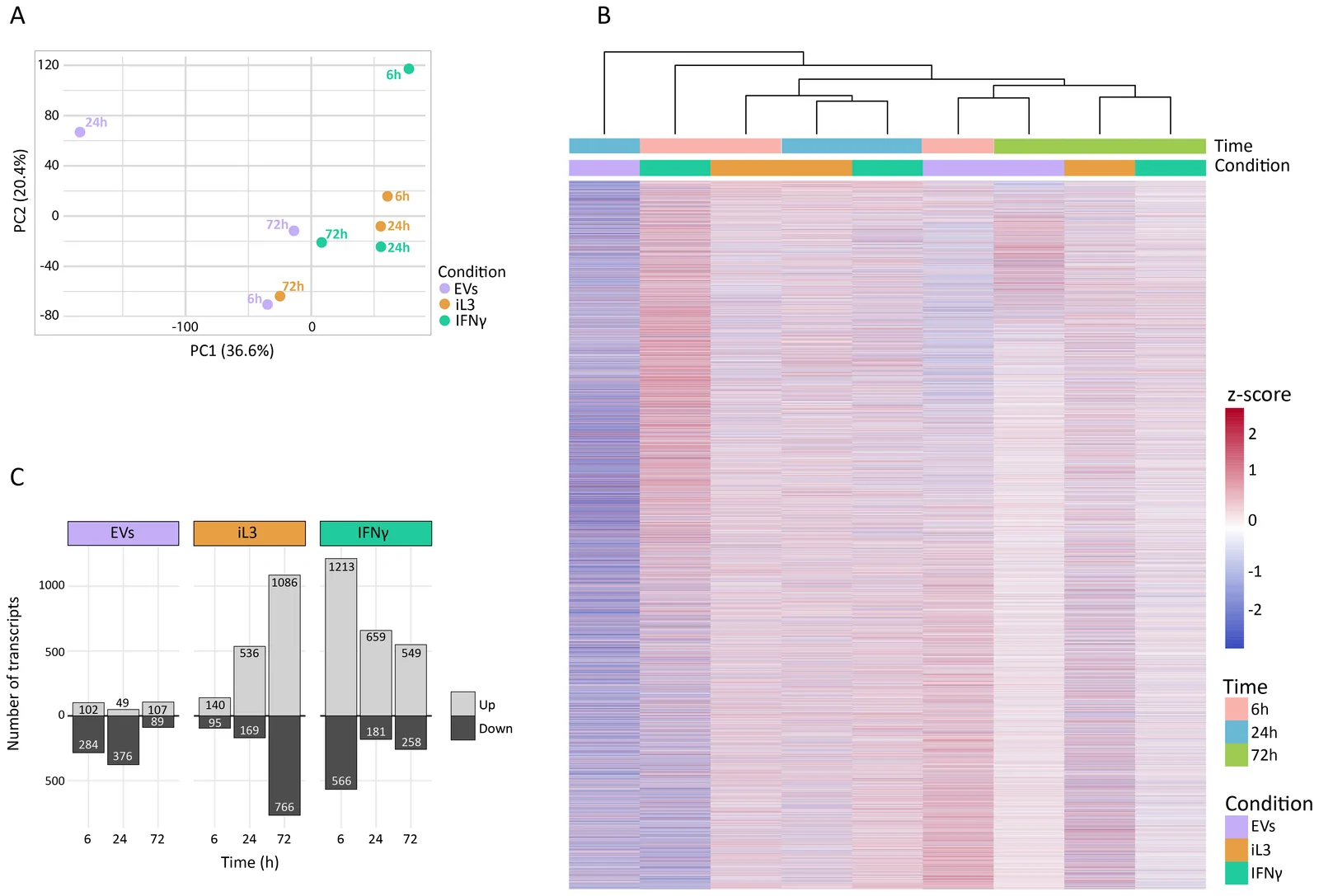
Modulation of mRNA expression in Caco-2 cells following exposure to the different stimuli. **(A)** PCA performed on mRNA FC relative to untreated cells. **(B)** HCA of mRNA relative abundances (i.e., FC of resting). Colour scale represents the z-score, i.e., scaled FC with mean=0 and standard deviation=1. **(C)** Number of differential mRNA in each condition compared to untreated resting cells, defined as having paired p-value <0.05 and |FC| ≥2.

Consistent with PCA results, HCA confirmed a distinct transcriptional signature associated with both treatment and time (**Figure 4B**). EV-treated cells at 24h formed a distinct cluster, clearly separated from all other samples. While iL3- and IFNγ-treated samples predominantly grouped according to the corresponding time point, EV-treated samples at 6h and 72h clustered together. Early IFNγ stimulation (6h) formed a separate branch from the remaining samples, reflecting the robust activation of a specific transcriptional response, however highlighting a close similarity to iL3 treated samples.

Differential expression analysis identified marked differences in the magnitude of the response of intestinal epithelial cells elicited by the three stimuli. Exposure to iL3 resulted in a progressive modulation over time, with 235,705 and 1,852 differentially expressed transcripts detected at 6, 24, and 72h, respectively. Notably, the late response to iL3 exposure was characterized by a substantial increase in both upregulated (1,086) and downregulated (766) genes. In contrast, EV stimulation elicited a comparatively modest response, with 386, 425 and 196 differentially expressed transcripts identified at 6, 24, and 72h, respectively, with a pick of down-modulated features (376) at 24h (**Figure 4C**; **Supplementary Figure S2**).

IFNγ induced the transcriptional perturbation at all investigated time points, with 1,779 differentially expressed transcripts at 6h, followed by 840 transcripts at 24h and 807 transcripts at 72h.

### 3.4 iL3 and their EVs modulate protein expression in Caco-2 cells

Proteomics analyses quantified 7,334 human proteins. Normalised mean intensities of the two technical replicates for each condition and the FC relative to untreated cells (i.e., treatment/resting) were first computed (**Supplementary_Table 2_Proteomics**). To evaluate the overall modulation in protein expression in response to the different stimuli (iL3, EVs, IFNγ) we performed a PCA analysis considering the FC (**Figure 5A**). The first two principal components accounted for 24.8% and 20.8% of the total variance, respectively. No clear separation between treatments was observed on either components; however, the three samples exposed to EVs at different time points clustered together, indicating that they were characterised by similar responses, which instead differed from those triggered by the exposure to iL3 or IFNγ. In these two latter conditions, similarity between samples was determined mainly by the time of exposure. Cells stimulated with larvae for 24h showed little modulation, hence very close to the PCA centre (0, 0). This trend was further confirmed by HCA. Unsupervised clustering clearly showed that EV samples clustered based on the stimulus, while iL3 and IFNγ clustered based on the time of exposure, independently of the stimulus (**Figure 5B**).

**Figure 5:**
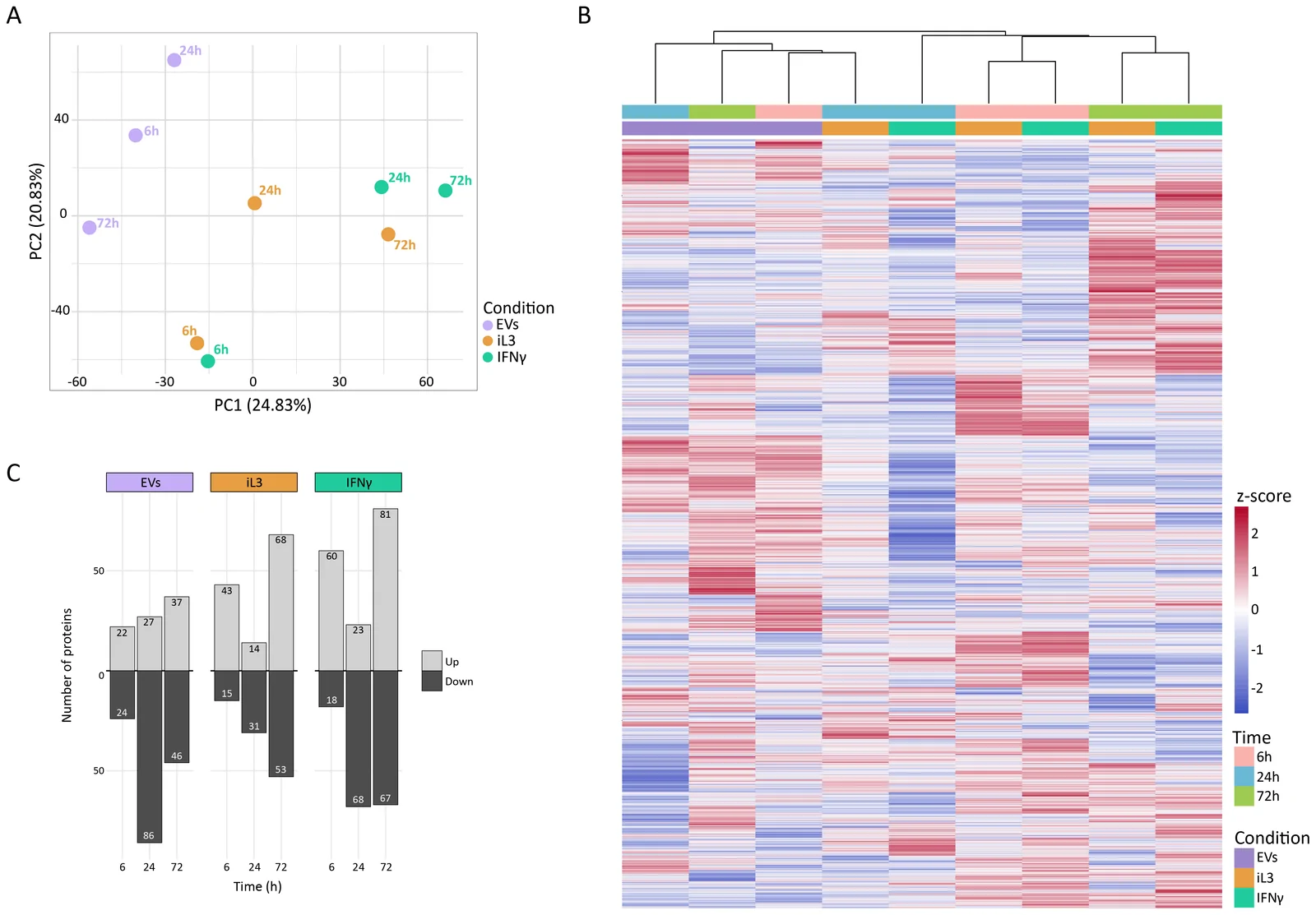
Modulation of protein expression in Caco-2 cells following exposure to the different stimuli. **(A)** PCA performed on the relative abundance of the proteins quantified by mass spectrometry with respect to resting cells (FC). **(B)** HCA of protein relative abundances (i.e., FC of resting). Colour scale represents the z-score, i.e., scaled FC with mean=0 and standard deviation=1. **(C)** Number of differential proteins in each condition compared to untreated resting cells, defined as having paired p-value <0.05 and |FC| ≥2.

The exposure to iL3 induced alterations in the protein expression of 58, 45 and 121 proteins at 6h, 24h and 72h, respectively. Exposure to EVs resulted in the modulation of 46, 113 and 82 proteins across the investigated time points, while IFNγ modulated 78, 91 and 148 proteins. EVs induced the strongest alterations at 24h, while larvae and IFNγ at 72h (**Figure 5C**; **Supplementary Figure S3**).

### 3.5 Pathway analysis reveals distinct temporal and biological responses induced by EVs and live larvae

To gain further insights into the biological processes modulated by the different stimuli, differentially expressed genes were analysed using IPA. Consistent with the clustering analyses, EVs and live larvae induced markedly different pathway modulation patterns over time, both at the mRNA and protein level (**Figures 6**, **7**).

**Figure 6:**
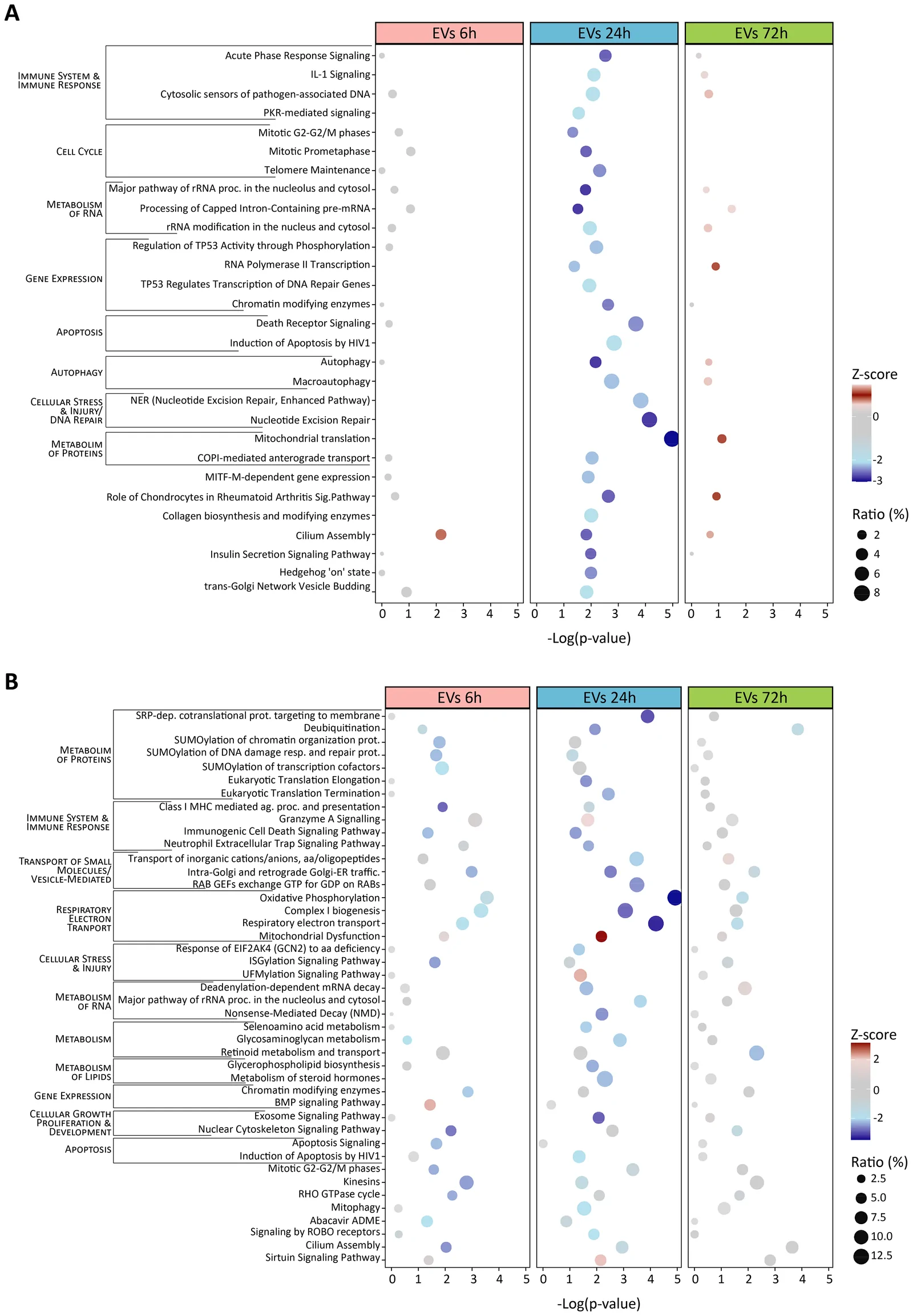
Pathway modulation in Caco-2 cells following exposure to EVs. **(A)** Modulation induced at the mRNA level over time. **(B)** Modulation induced at the protein level over time. Colour scale represents pathway activation Z-score, indicative of the overall pathway activation/inhibition state; circle size (% Ratio) represents the percentage of molecules of the pathway identified in the dataset; -Log(p-value) indicates the probability of association of molecules from the dataset with the canonical pathway by random chance alone (right-tailed Fisher’s Exact Test).

**Figure 7:**
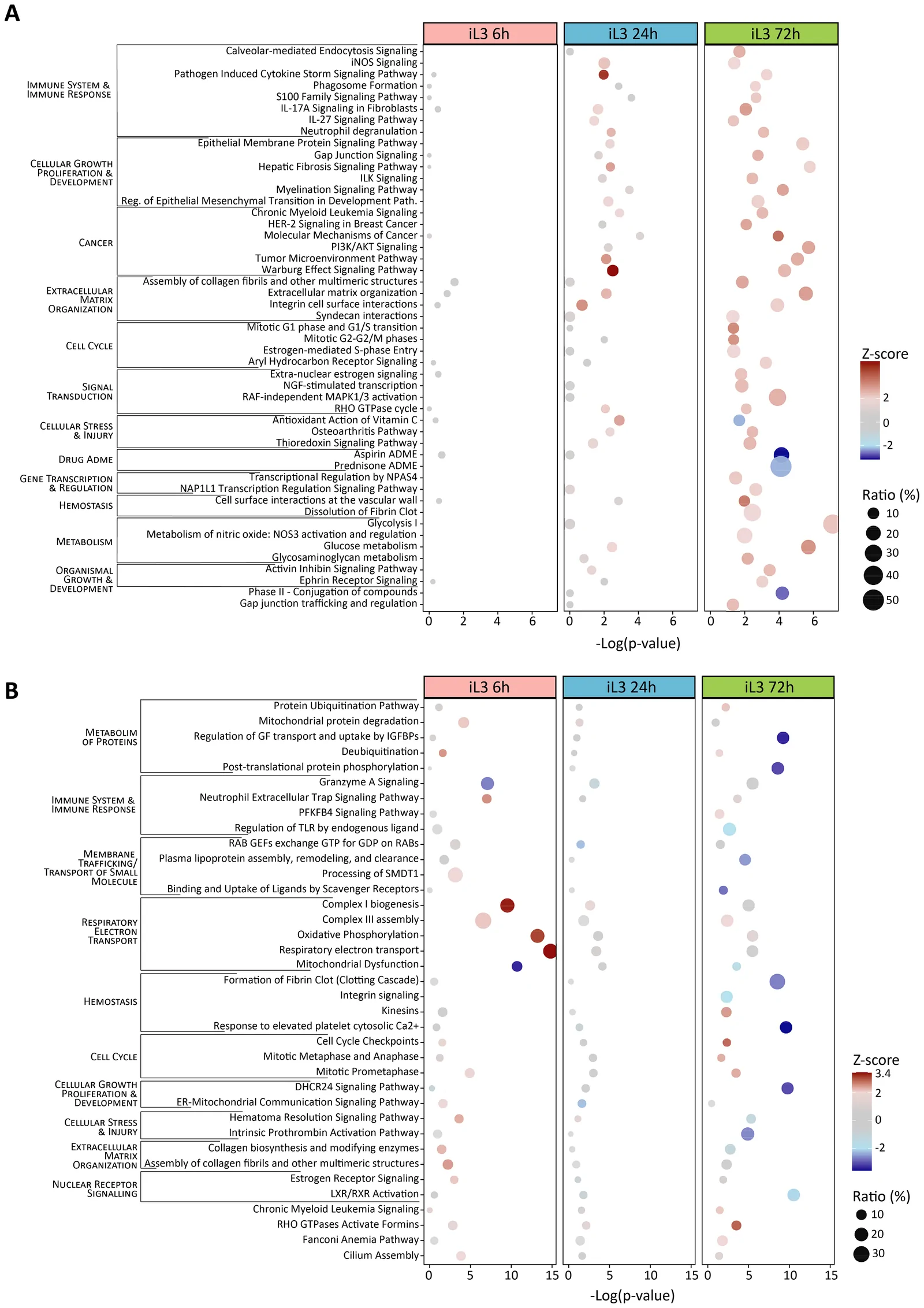
Pathway modulation in Caco-2 cells following exposure to iL3. **(A)** Modulation induced at the mRNA level over time. **(B)** Modulation induced at the protein level over time. Colour scale represents pathway activation Z-score, indicative of the overall pathway activation/inhibition state; circle size (% Ratio) represents the percentage of molecules of the pathway identified in the dataset; -Log(p-value) indicates the probability of association of molecules from the dataset with the canonical pathway by random chance alone (right-tailed Fisher’s Exact Test).

In Caco-2 cells exposed to EVs, a total of 67 pathways were represented as significantly modulated, of which 29 modulated at transcriptional level (**Figure 6A**) and 43 at protein level (**Figure 6B**). In agreement with the number of modulated proteins, pathway enrichment was most pronounced at 24h, whereas only a limited number of pathways were significantly represented at 6h and 72h. Most significantly enriched pathways displayed negative activation scores, indicating an overall inhibitory effect of EV exposure on host-cell functions.

Despite the limited overlap at the individual pathway level, both datasets converged on common biological processes. Enriched pathways were classified into 13 categories based on their association with biological processes. Of these, 8 pathway categories were represented at the transcriptome while 11 at the proteome level, 6 of which were shared between the two omics datasets. Common pathways categories included immune signalling, metabolism of RNA and proteins, gene expression regulation, apoptosis, cellular stress and injury.

At the transcriptomic level, pathway modulation included the inhibition of RNA biogenesis and processing pathways - including rRNA processing and modification, pre-mRNA processing and RNA Polymerase II transcription - together with pathways involved in DNA repair and cellular homeostasis, such as nucleotide excision repair, TP53-mediated DNA repair, telomere maintenance, autophagy and macroautophagy. Mitochondrial translation represented the most significantly enriched pathway at 24h and showed the strongest predicted inhibition (z-score = -3).

Immune-related pathways were also affected: acute phase response signalling, IL-1 signalling and cytosolic sensors of pathogen-associated DNA were predicted to be inhibited particularly at 24h, suggesting suppression of epithelial innate immune responses following EV exposure.

At the protein level, EVs induced a particularly strong pathway modulation at 24h (27 modulated pathways), even though a robust response was already evident at 6h post-exposure, with 16 significantly modulated pathways. At 72h, although 83 proteins were significantly differentially expressed compared to untreated cells, this translated into minimal functional impact, with only a single pathway significantly affected according to our criteria.

Overall, proteomic analysis revealed a predominant inhibition of pathways involved in metabolism of protein, RNA or other small molecules, as well as with vesicle mediated transport and respiratory electron transport. Consistent with mRNA findings, pathways associated with the immune response were modulated also at the protein level, starting as early as 6h post-exposure and peaking at 24h; however, more variability was observed since three pathways were inhibited (Class I MHC mediated antigen processing and presentation, immunogenic cell death pathways, NET signalling pathway), while granzyme A signalling was predicted as activated. Pathways associated with respiratory electron transport were the most significantly inhibited by EVs at the protein level, reaching z-score < -3.

In Caco-2 cells exposed to live larvae, a total of 84 significantly enriched pathways were identified, including 49 detected at the transcriptomic level (**Figure 7A**) and 37 at the proteomic level (**Figure 7B**), with two pathways shared between the two datasets. These pathways were grouped into 16 functional categories, including 12 represented in the transcriptomic dataset and 10 in the proteomic one, 6 of which were common to both omics layers.

Transcriptomic pathway analysis revealed a progressive increase in both the number and significance of enriched pathways over time, with the strongest response observed at 72h. In contrast to EV-treated cells, most pathways identified following larval exposure were predicted to be activated, indicating sustained host-cell activation.

The most significantly enriched pathways were associated with innate and inflammatory responses, including pathogen-induced cytokine storm signaling, IL-17A signaling, IL-27 signaling, iNOS signaling, phagosome formation and neutrophil degranulation.

Several pathways involved in epithelial remodelling and host–parasite interactions were also activated, including epithelial membrane protein signaling, integrin cell surface interactions, ILK signaling, regulation of epithelial-to-mesenchymal transition and extracellular matrix organization. Consistently, extracellular matrix remodelling pathways, including collagen assembly and organisation, showed increased representation and activation over time. Activation of pathways associated with cell proliferation, survival and metabolic adaptation was also evident, including PI3K/AKT signaling, molecular mechanisms of cancer, tumor microenvironment pathway, RHO GTPase signalling, glycolysis and glucose metabolism. Together, these findings indicate increased energetic demand and extensive transcriptional reprogramming following larval exposure.

Similar to the modulation observed at the mRNA level, Caco-2 cells exposed to larvae also showed the strongest modulation also in protein expression at 72h, although a marked change was already detectable as early as 6h post-exposure. Compared to cells exposed to EVs, higher variability was observed in response to larvae. Indeed, although the most affected pathways included metabolism of proteins and the immune response, no clear trend towards activation or inhibition was observed. Nevertheless, it is interesting to note that granzyme A signalling and NET formation pathways displayed an opposite activation profile compared to EVs.

Proteomic analysis further supported the progressive activation observed at the transcriptomic level. In particular, pathways associated with respiratory electron transfer, oxidative phosphorylation, mitochondrial biogenesis, and cell cycle became increasingly represented over time, as opposed to what was observed with EVs. Modulation of pathways involved in ubiquitination/deubiquitination, intracellular trafficking and extracellular matrix organisation further suggests extensive cellular remodelling in response to larval exposure.

Cells exposure to IFNγ induced a significant activation of interferon signalling pathways at both mRNA and protein level, at all investigated time points (**Supplementary Figure S5**).

## 4 Discussion

Helminth parasites are master manipulators of the host immune system. Indeed, natural infections often induce mechanisms of immune tolerance that are associated with both chronic persistence, and parasite expulsion and clearance (Maizels et al., 2018). The peculiar capacity of auto-infection of *S. stercoralis*, however, hampers parasite clearance and might favour immune-tolerance (King and Li, 2018). Importantly, parasite-derived immunomodulatory factors, including ESP and EVs, contribute to the establishment of this tolerance state (Zumbado-Salas et al., 2025). Recently, we have shown that *S. stercoralis* infective larvae isolated from clinical samples release extracellular vesicles containing parasite-derived small-RNAs (Deiana et al., 2026). In the present study we used an *in vitro* system combined with multi-omics approaches to investigate the modulation induced by *S. stercoralis* iL3 and their EVs in human intestinal epithelial cells to gain new insights into the local host-pathogen interaction during strongyloidiasis. Our results revealed that different responses are triggered by the two stimuli over time. Indeed, live larvae induced cellular activation and a pro-inflammatory-like profile that peaked 72h post-exposure, while EVs displayed a quicker effect with modulation in mRNA and protein expression mostly 24h post-treatment and leading to a predominant inhibition of cell activation, metabolism and immune response.

Intestinal epithelial cells play a pivotal role in the pathogenesis of helminth infections, acting both as a physical barrier and as sensors and effectors of host defence against invading pathogens (Coakley and Harris, 2020). Together with other cell types, epithelial cells release alarmins – namely IL-25, IL-33 and TSLP – which trigger the activation of ILC2 and type 2 CD4^+^ T helper cells (Oyesola et al., 2021), driving the secretion of type 2 cytokines. These responses combined with alterations in the intestinal epithelium, ultimately lead to the “weep and sweep” response and parasite expulsion (Baska and Norbury, 2022). Here, we evaluated the release of alarmins, which are involved in initiating the intestinal immune response (Coakley and Harris, 2020), by exposing Caco-2 cells to iL3 and to iL3-EVs. However, IL-25, IL-33 and TSLP were not detected in the conditioned medium in our experiments, in agreement with previous studies showing the presence of alarmin transcripts but not the corresponding proteins in this cell line (Benede et al., 2022). A significant release of pro-inflammatory chemokines, i.e., IL-8 and CXCL10, was instead observed in response to live *S. stercoralis* iL3, as early as 24h post-exposure. EVs induced the release of CXCL10 only, although to a lesser extent compared to live larvae. At this stage it would be premature to hypothesise weather this different profile is associated with a mitigated effect mediated by EVs or with their potential involvement in immune-escaping. Contrasting results are, in fact, reported in the literature regarding the effects of helminths and their derived products on the intestinal epithelium. For instance, inhibition of IL-6 and IL-8 by *Anisakis* L3 and their EVs was reported in Caco-2 cells (Bellini et al., 2022), while *Trichuris muris* ESP stimulated the production of pro-inflammatory cytokines in murine intestinal epithelial cells (deSchoolmeester et al., 2006). These apparent inconsistencies might be ascribable to the different biology and living niche of diverse helminths (Baska and Norbury, 2022) and to the variable settings/designs of experimental studies.

To evaluate host-cell response in a broader context, we used multi-omics approaches and analysed gene expression modulation in treated Caco-2 cells. The ability of our system to respond to external stimuli was assessed and confirmed by treating cells with IFNγ used as a pro-inflammatory control.

Parasite-derived EVs down-modulated IEC activity both at the mRNA and protein level, particularly at 24h. This early response to EVs affected protein metabolism, gene expression regulation, cell cycle and the immune response. Proteins associated with SUMOylation, a dynamic post-translational modification involved in gene expression regulation acting on transcription factors and chromatin organisation (Tharuka et al., 2025), were overall down-modulated in our dataset. Although not studied yet in the context of the host response to helminth infections, suppression of SUMOylation pathways by bacterial infections was proposed as a mechanism to impair the host immune response (Xu et al., 2025). In agreement with this, at 6h, we observed decreased antigen presentation and immunogenic cell death, with the latter being associated with the release of damage-associated molecular patterns (DAMPs) that will be recognised by pattern recognition receptors (PRRs) on immune cells as part of a regulated cell death. Although mainly studied in cancer (Kroemer et al., 2022), evidence of the association of immunogenic cell death with infectious diseases is being collected (Zhang et al., 2024). This earlier response was followed by reduced inflammatory signalling and immune recognition at 24h post-exposure to EVs. At the protein level, pathways associated with respiratory electron transport were also down-modulated following exposure to EVs across the different time points, although statistical significance was observed only at 24h. This suggests that EVs exposure might impair mitochondrial activity and, overall, interfere with the ability of the host immune system to recognise the invading pathogens. As already observed for other helminths (Drurey and Maizels, 2021), EVs could thus represent one of the earliest signal of interaction with the host, contributing to remodelling of the intestinal tissue to favour the establishment of the infection. While the molecular mechanisms underlying the observed changes in gene expression mediated by EVs remain unclear, recent characterisation of *S. stercoralis* iL3-derived EVs demonstrated the presence of parasite small RNAs predicted to target human immune-related genes (Deiana et al., 2026), suggesting a potential contribution of EV-associated RNA cargo to the epithelial response observed here.

Our omics data suggested that live larvae exerted the strongest effect on target cells at a later time point compared to EVs, i.e., at 72h. Moreover, opposite to EVs, iL3 mainly induced cell activation, in agreement with the important role of epithelial cells in sensing and recognising helminth pathogens (Coakley and Harris, 2020; Karo-Atar et al., 2023). Gene expression modulation at 72h mainly affected pathways associated with the immune response, cellular growth, proliferation and cell cycle, membrane trafficking and extracellular organisation. Importantly, most of these pathways were activated in response to iL3, particularly those associated with the immune response, cell cycle and cell activity, suggesting that a prolonged exposure to live larvae stimulate epithelial defence mechanisms. In agreement with the increased cellular activity, iL3 also induced an early (6h) activation of respiratory electron transport at the protein level. It is worth noticing that overall, iL3 showed a more similar profile to IFNγ - here used as a positive control to assess Caco-2 reactivity to a pro-inflammatory stimulus - rather than to EVs. This might indicate that, at least in a short period of time, iL3 trigger cell activation with a profile similar to a pro-inflammatory stimulus, while EV seem to down modulate cell activity and responsiveness.

Our study has some limitations that need to be mentioned. Studies with *Nippostrongylus brasiliensis* and *Heligmosomoides polygyrus* have revealed that the type 2 immunity that develops following helminth infection results from multiple events, such as Tuft and goblet cell activation, tissue damage and pathogen-associated molecular patterns (PAMPs) recognition (Sorobetea et al., 2018; Coakley and Harris, 2020; Campillo Poveda et al., 2023). Although the specific role of the intestinal epithelium in human strongyloidiasis remains unexplored, it is clear that different cell types are important in orchestrating the host response to helminth parasites. Moreover, it has been shown that intestinal epithelial cells derived from induced pluripotent stem cells (iPSC) displayed a stronger pro-inflammatory response following inflammatory challenge compared to Caco-2 cells (Janssen et al., 2024), further highlighting the importance of the choice of the target cell type to be investigated. In our study we explored the interaction between *S. stercoralis* and the gut using a simplistic setting based on Caco-2 cells. Despite the lack of complexity, our results give new insights into the local interaction between clinically-isolated infective larvae and the epithelial cells of the gut, and provide the first evidence of a potential role for iL3-derived EVs in the local host-pathogen interaction in strongyloidiasis. Our observations obtained using enriched EVs pave the way for more in-depth investigations of the entire iL3-derived ESP, encompassing both EVs and soluble factors.

Moreover, as observed for *N. brasiliensis* - which establishes the infection in the small intestine following a similar migration route (Galan-Puchades et al., 2025) - other cell types encountered in the different anatomical sites during migration might play a critical role in shaping the host response to the infection. Similarly, interactions between adult female worms and the intestinal epithelium should be taken into account, given that they reside in the small intestine *in vivo*. However, obtaining parasitic females from infected individuals remains particularly challenging. Further in-depth investigations are warranted to elucidate these aspects.

In this work we focused our investigations on alterations in gene expression, affecting mRNA, proteins and cytokine release. Future studies should evaluate the effects on the integrity of the epithelial barrier, although models involving multiple cell types might be more suitable.

In order to increase the biological relevance of our findings, in our study we investigated iL3 isolated from a clinical sample. Since only one isolate was employed, our results should be confirmed using additional independent biological replicates, in order to evaluate the biological reproducibility of our findings and to investigate potential isolate/genotype-dependent effects. However, obtaining sufficient numbers of viable *S. stercoralis* infective larvae from clinical samples remains technically challenging and largely dependent on sample availability and parasite burden. Therefore, the present study should be interpreted as an exploratory investigation obtained with only one clinical isolate, and aimed at generating hypotheses on the intestinal host-parasite interaction using strains of *S. stercoralis* originating from clinical samples. Despite these limitations, the use of a clinical isolate represents a relevant strength of the present model, as it provides an experimental system based on parasites directly derived from an infected patient, rather than on laboratory-adapted strains.

Similarly to previous studies (Stryinski et al., 2025), under our experimental conditions we observed partly distinct biological effects mediated by EVs and live larvae. Although our data do not provide mechanistic explanations for these differences, it can be speculated that EVs represent one of the mechanisms employed by *S. stercoralis* to interact with the host and do not necessarily recapitulate the effects of the intact parasite. Indeed, EVs represent an important mechanism for release and delivery of bioactive molecules (Drurey and Maizels, 2021). Further comprehensive investigations are required to confirm our findings and to establish the relationship between the exposure doses of larvae and EVs and the biological effects observed.

In conclusion, the interaction between helminth parasites and the intestinal epithelium is central for triggering the local immune response and for the determination of the fate of the infection. Most of our current knowledge regarding immunity to helminth parasites and their interaction with the host at the intestinal level has been collected using model helminth parasites (i.e., *N. brasiliensis*, *H. polygyrus* and *T. muris*) (Michla and Wilhelm, 2022). Although essential to widen our understanding of these mechanisms, not all the observations collected using those models can be generalised, since each parasite has its own peculiarity. This aspect is particularly relevant for *S. stercoralis* due to its unique capacity of autoinfection in the human host. Here, we describe for the first time how *S. stercoralis* iL3 and their EVs act on intestinal epithelial cells. Our results revealed that effects mediated by EVs not necessarily recapitulate those induced by live larvae. Indeed, while live larvae seem to promote cellular activation and host response, EVs down-modulate cell metabolism and immune response. Although speculative, our results open the question on whether, as already shown for other helminth parasites (Dagenais et al., 2021; Drurey and Maizels, 2021), *S. stercoralis* EVs might be part of an evasion strategy of this parasite to promote its survival within the host.

## Data Availability

The datasets presented in this study can be found in online repositories. NGS raw data have been deposited in the European Nucleotide Archive (ENA) under the study accession number PRJEB123878. The mass spectrometry proteomics data have been deposited to the ProteomeXchange Consortium via the PRIDE (Perez-Riverol et al., 2019) partner repository with the dataset identifier PXD082958.

## References

[B1] Allen J. E. Wynn T. A. (2011). Evolution of th2 immunity: a rapid repair response to tissue destructive pathogens. PLoS Pathog. 7, e1002003. doi: 10.1371/journal.ppat.1002003 21589896 PMC3093361

[B2] Barratt J. L. N. Lane M. Talundzic E. Richins T. Robertson G. Formenti F. . (2021). Correction: a global genotyping survey of strongyloides stercoralis and strongyloides fuelleborni using deep amplicon sequencing. PLoS Negl.Trop. Dis. 15, e0009538. doi: 10.1371/journal.pntd.0009538 31525192 PMC6762204

[B3] Barroso M. Salvador F. Sanchez-Montalva A. Bosch-Nicolau P. Molina I. (2019). Strongyloides stercoralis infection: a systematic review of endemic cases in spain. PLoS Negl.Trop. Dis. 13, e0007230. doi: 10.1371/journal.pntd.0007230 30860995 PMC6413904

[B4] Baska P. Norbury L. J. (2022). The role of the intestinal epithelium in the "weep and sweep" response during gastro-intestinal helminth infections. Anim. (Basel) 12, 1–13. doi: 10.3390/ani12020175 35049796 PMC8772803

[B5] Bellini I. Scribano D. Sarshar M. Ambrosi C. Pizzarelli A. Palamara A. T. . (2022). Inflammatory response in caco-2 cells stimulated with anisakis messengers of pathogenicity. Pathogens 11, 2–13. doi: 10.3390/pathogens11101214 36297271 PMC9611079

[B6] Benede S. Perez-Rodriguez L. Molina E. (2022). Caco-2 cell response induced by peptides released after digestion of heat-treated egg white proteins. Foods 11, 1–8. doi: 10.3390/foods11223566 36429158 PMC9689089

[B7] Bouchery T. Le Gros G. Harris N. (2019). Ilc2s-trailblazers in the host response against intestinal helminths. Front. Immunol. 10, 623. doi: 10.3389/fimmu.2019.00623 31019505 PMC6458269

[B8] Breloer M. Linnemann L. (2024). Strongyloides ratti infection in mice: immune response and immune modulation. Philos. Trans. R. Soc Lond. B. Biol. Sci. 379, 20220440. doi: 10.1098/rstb.2022.0440 38008111 PMC10676808

[B9] Buck A. H. Coakley G. Simbari F. McSorley H. J. Quintana J. F. Le Bihan T. . (2014). Exosomes secreted by nematode parasites transfer small rnas to mammalian cells and modulate innate immunity. Nat. Commun. 5, 5488. doi: 10.1038/ncomms6488 25421927 PMC4263141

[B10] Buonfrate D. Bisanzio D. Giorli G. Odermatt P. Furst T. Greenaway C. . (2020). The global prevalence of strongyloides stercoralis infection. Pathogens 9, 1–9. doi: 10.3390/pathogens9060468 32545787 PMC7349647

[B11] Buonfrate D. Salas-Coronas J. Munoz J. Maruri B. T. Rodari P. Castelli F. . (2019). Multiple-dose versus single-dose ivermectin for strongyloides stercoralis infection (strong treat 1 to 4): a multicentre, open-label, phase 3, randomised controlled superiority trial. Lancet Infect. Dis. 19, 1181–1190. doi: 10.1016/S1473-3099(19)30289-0 31558376

[B12] Buonfrate D. Tamarozzi F. Paradies P. Watts M. R. Bradbury R. S. Bisoffi Z. (2022). The diagnosis of human and companion animal strongyloides stercoralis infection: challenges and solutions. a scoping review. Adv. Parasitol. 118, 1–84. doi: 10.1016/bs.apar.2022.07.001 36088083

[B13] Campillo Poveda M. Britton C. Devaney E. McNeilly T. N. Gerbe F. Jay P. . (2023). Tuft cells: detectors, amplifiers, effectors and targets in parasite infection. Cells 12, 1–12. doi: 10.3390/cells12202477 37887321 PMC10605326

[B14] Castelletto M. L. Akimori D. Patel R. Schroeder N. E. Hallem E. A. (2024). Introduction to strongyloides stercoralis anatomy. J. Nematol. 56, 20240019. doi: 10.2478/jofnem-2024-0019 38855080 PMC11162604

[B15] Coakley G. Harris N. L. (2020). The intestinal epithelium at the forefront of host-helminth interactions. Trends Parasitol. 36, 761–772. doi: 10.1016/j.pt.2020.07.002 32713764

[B16] Coakley G. McCaskill J. L. Borger J. G. Simbari F. Robertson E. Millar M. . (2017). Extracellular vesicles from a helminth parasite suppress macrophage activation and constitute an effective vaccine for protective immunity. Cell Rep. 19, 1545–1557. doi: 10.1016/j.celrep.2017.05.001 28538175 PMC5457486

[B17] Dagenais M. Gerlach J. Q. Wendt G. R. Collins J. J. 3rd Atkinson L. E. . (2021). Analysis of schistosoma mansoni extracellular vesicles surface glycans reveals potential immune evasion mechanism and new insights on their origins of biogenesis. Pathogens 10, 1–15. doi: 10.3390/pathogens10111401 34832557 PMC8617790

[B18] Deiana M. Veschetti L. Reynolds K. Hunt V. L. Padovani N. Manfredi M. . (2026). Molecular characterisation of extracellular vesicles released by strongyloides stercoralis infective larvae isolated from a clinical sample. Parasit Vectors 19, 1–11. doi: 10.1186/s13071-026-07484-0 42237397 PMC13330409

[B19] deSchoolmeester M. L. Manku H. Else K. J. (2006). The innate immune responses of colonic epithelial cells to trichuris muris are similar in mouse strains that develop a type 1 or type 2 adaptive immune response. Infect. Immun. 74, 6280–6286. doi: 10.1128/IAI.01609-05 17057095 PMC1695505

[B20] Drurey C. Maizels R. M. (2021). Helminth extracellular vesicles: interactions with the host immune system. Mol. Immunol. 137, 124–133. doi: 10.1016/j.molimm.2021.06.017 34246032 PMC8636279

[B21] Duque-Correa M. A. Schreiber F. Rodgers F. H. Goulding D. Forrest S. White R. . (2020). Development of caecaloids to study host-pathogen interactions: new insights into immunoregulatory functions of trichuris muris extracellular vesicles in the caecum. Int. J. Parasitol. 50, 707–718. doi: 10.1016/j.ijpara.2020.06.001 32659277 PMC7435689

[B22] Fleitas P. E. Travacio M. Marti-Soler H. Socias M. E. Lopez W. R. Krolewiecki A. J. (2020). The strongyloides stercoralis-hookworms association as a path to the estimation of the global burden of strongyloidiasis: a systematic review. PLoS Negl.Trop. Dis. 14, e0008184. doi: 10.1371/journal.pntd.0008184 32282827 PMC7188296

[B23] Galan-Puchades M. T. Gomez-Samblas M. Trelis M. Osuna A. Bueno-Mari R. Saez-Duran S. . (2025). New data on the life cycle of nippostrongylus brasiliensis (travassos 1914) (nematoda: heligmosomidae): development of eggs and larval stages in the intestine of naturally infected rattus norvegicus (berkenhout 1769). Parasitol. Res. 124, 20. doi: 10.1007/s00436-025-08462-8 39909911 PMC11799119

[B24] Gordon C. A. Utzinger J. Muhi S. Becker S. L. Keiser J. Khieu V. . (2024). Strongyloidiasis. Nat. Rev. Dis. Primers 10, 6. doi: 10.1038/s41572-023-00490-x 38272922

[B25] Hartmann W. Heepmann L. Linnemann L. Licona-Limon P. Colomb F. Frangova T. . (2026). Dynamics of interleukin-9 producing lymphocytes in strongyloides ratti-infected mice. Pathogens 15, 1–14. doi: 10.3390/pathogens15030257 41901710 PMC13029138

[B26] Heepmann L. Hartmann W. Linnemann L. Dorken S. Viebrock B. Orinska Z. . (2026). CD160-competent ilc2 are crucial for the ejection of intestinal helminths by the innate immune system. Mucosal Immunol. 19, 100344. doi: 10.1016/j.mucimm.2026.04.005 41974253

[B27] Inclan-Rico J. M. Siracusa M. C. (2018). First responders: innate immunity to helminths. Trends Parasitol. 34, 861–880. doi: 10.1016/j.pt.2018.08.007 30177466 PMC6168350

[B28] Janssen A. W. F. van der Lugt B. Duivenvoorde L. P. M. Vos A. P. Bastiaan-Net S. Tomassen M. M. M. . (2024). Comparison of ipsc-derived human intestinal epithelial cells with caco-2 cells and human *in vivo* data after exposure to lactiplantibacillus plantarum wcfs1. Sci. Rep. 14, 26464. doi: 10.1038/s41598-024-74802-w 39488516 PMC11531526

[B29] Karo-Atar D. Gregorieff A. King I. L. (2023). Dangerous liaisons: how helminths manipulate the intestinal epithelium. Trends Parasitol. 39, 414–422. doi: 10.1016/j.pt.2023.03.012 37076358

[B30] King I. L. Li Y. (2018). Host-parasite interactions promote disease tolerance to intestinal helminth infection. Front. Immunol. 9, 2128. doi: 10.3389/fimmu.2018.02128 30298071 PMC6160735

[B31] Kramer A. Green J. Pollard J. Jr. Tugendreich S. (2014). Causal analysis approaches in ingenuity pathway analysis. Bioinformatics 30, 523–530. doi: 10.1093/bioinformatics/btt703 24336805 PMC3928520

[B32] Kroemer G. Galassi C. Zitvogel L. Galluzzi L. (2022). Immunogenic cell stress and death. Nat. Immunol. 23, 487–500. doi: 10.1038/s41590-022-01132-2 35145297

[B33] Maizels R. M. Smits H. H. McSorley H. J. (2018). Modulation of host immunity by helminths: the expanding repertoire of parasite effector molecules. Immunity 49, 801–818. doi: 10.1016/j.immuni.2018.10.016 30462997 PMC6269126

[B34] Maizels R. M. Yazdanbakhsh M. (2003). Immune regulation by helminth parasites: cellular and molecular mechanisms. Nat. Rev. Immunol. 3, 733–744. doi: 10.1038/nri1183 12949497

[B35] Meiners J. Reitz M. Rudiger N. Turner J. E. Heepmann L. Rudolf L. . (2020). IL-33 facilitates rapid expulsion of the parasitic nematode strongyloides ratti from the intestine via ilc2- and il-9-driven mast cell activation. PLoS Pathog. 16, e1009121. doi: 10.1371/journal.ppat.1009121 33351862 PMC7787685

[B36] Michla M. Wilhelm C. (2022). Food for thought - ilc metabolism in the context of helminth infections. Mucosal Immunol. 15, 1234–1242. doi: 10.1038/s41385-022-00559-y 36045216 PMC9705246

[B37] Natoli M. Leoni B. D. D'Agnano I. Zucco F. Felsani A. (2012). Good caco-2 cell culture practices. Toxicol. Vitro 26, 1243–1246. doi: 10.1016/j.tiv.2012.03.009 22465559

[B38] Oyesola O. O. Shanahan M. T. Kanke M. Mooney B. M. Webb L. M. Smita S. . (2021). PGD2 and CRTH2 counteract type 2 cytokine-elicited intestinal epithelial responses during helminth infection. J. Exp. Med. 218, 1–20. doi: 10.1084/jem.20202178 34283207 PMC8294949

[B39] Perez-Riverol Y. Csordas A. Bai J. Bernal-Llinares M. Hewapathirana S. Kundu D. J. . (2019). The pride database and related tools and resources in 2019: improving support for quantification data. Nucleic Acids Res. 47, D442–D450. doi: 10.1093/nar/gky1106 30395289 PMC6323896

[B40] Shi Y. He C. Ma C. Yu T. Cong Y. Cai W. . (2018). Smad nuclear interacting protein 1 (snip1) inhibits intestinal inflammation through regulation of epithelial barrier function. Mucosal Immunol. 11, 835–845. doi: 10.1038/mi.2017.95 29426045

[B41] Soblik H. Younis A. E. Mitreva M. Renard B. Y. Kirchner M. Geisinger F. . (2011). Life cycle stage-resolved proteomic analysis of the excretome/secretome from strongyloides ratti--identification of stage-specific proteases. Mol. Cell. Proteomics 10, M111 010157. doi: 10.1074/mcp.M111.010157 21964353 PMC3237078

[B42] Sorobetea D. Svensson-Frej M. Grencis R. (2018). Immunity to gastrointestinal nematode infections. Mucosal Immunol. 11, 304–315. doi: 10.1038/mi.2017.113 29297502

[B43] Sotillo J. Robinson M. W. Kimber M. J. Cucher M. Ancarola M. E. Nejsum P. . (2020). The protein and microrna cargo of extracellular vesicles from parasitic helminths - current status and research priorities. Int. J. Parasitol. 50, 635–645. doi: 10.1016/j.ijpara.2020.04.010 32652128

[B44] Stryinski R. Fiedorowicz E. Mateos J. Andronowska A. Lopienska-Biernat E. Carrera M. (2025). Exploring the exoproteome of the parasitic nematode anisakis simplex (s. s.) and its impact on the human host - an *in vitro* cross-talk proteomic approach. Front. Immunol. 16, 1509984. doi: 10.3389/fimmu.2025.1509984 39963139 PMC11830668

[B45] Tharuka M. D. N. Courelli A. S. Chen Y. (2025). Immune regulation by the sumo family. Nat. Rev. Immunol. 25, 608–620. doi: 10.1038/s41577-025-01155-4 40108400

[B46] Tiberti N. Bertoli G. Mazzi C. Vezzelli E. Zanzoni S. Alladio F. . (2026). Profiling of circulating immune factors and host-derived extracellular vesicles in response to bacterial or malaria acute fever. Infection 54, 443–458. doi: 10.1007/s15010-025-02694-7 41258581 PMC12864194

[B47] Trelis M. Galiano A. Bolado A. Toledo R. Marcilla A. Bernal D. (2016). Subcutaneous injection of exosomes reduces symptom severity and mortality induced by echinostoma caproni infection in balb/c mice. Int. J. Parasitol. 46, 799–808. doi: 10.1016/j.ijpara.2016.07.003 27590846

[B48] White R. Sotillo J. Ancarola M. E. Borup A. Boysen A. T. Brindley P. J. . (2023). Special considerations for studies of extracellular vesicles from parasitic helminths: a community-led roadmap to increase rigour and reproducibility. J. Extracell. Vesicles 12, e12298. doi: 10.1002/jev2.12298 36604533 PMC9816087

[B49] Xu Y. Ma X. Wu Z. Huang R. Liao C. Huang D. . (2025). Host sumoylation in bacterial infections and immune defense mechanisms. Front. Microbiol. 16, 1621137. doi: 10.3389/fmicb.2025.1621137 40641872 PMC12241045

[B50] Yasuda K. Matsumoto M. Nakanishi K. (2014). Importance of both innate immunity and acquired immunity for rapid expulsion of s. venezuelensis. Front. Immunol. 5, 118. doi: 10.3389/fimmu.2014.00118 24678315 PMC3958730

[B51] Yeshi K. Ruscher R. Loukas A. Wangchuk P. (2022). Immunomodulatory and biological properties of helminth-derived small molecules: Potential applications in diagnostics and therapeutics. Front. Parasitol. 1, 984152. doi: 10.3389/fpara.2022.984152 39816468 PMC11731824

[B52] Zhang Y. H. Xie L. H. Li J. Qi Y. W. Shi J. J. (2024). Classification and clinical significance of immunogenic cell death-related genes in Plasmodium falciparum infection determined by integrated bioinformatics analysis and machine learning. Malar. J. 23, 48. doi: 10.1186/s12936-024-04877-3 38360586 PMC10868002

[B53] Zumbado-Salas M. Rojas A. Maizels R. M. Mora J. (2025). Cross-species immunomodulation by zoonotic helminths: The roles of excretory/secretory products and extracellular vesicles. Curr. Top. Membr. 96, 47–84. doi: 10.1016/bs.ctm.2025.08.001 41248960

