# Supplementary material for: In vitro investigation of the host-pathogen interaction in human strongyloidiasis at the intestinal level
Source: Front Cell Infect Microbiol. 2026 Oct 28;16:1981291. doi: 10.3389/fcimb.2026.1981291 (PMC13751453; doi:10.3389/fcimb.2026.1981291)

Supplementary Material

Table S1. Detailed number of *S. stercoralis* iL3 used to stimulate Caco-2 cells in each experiment.

**Replicate**	**6h**	**24h**	**72h**
#1	19	9	11
#2	17	7	8
#3	15	30	11
#4	13	12	19

Table S2. Median fluorescence intensity values (MFI) obtained for the cytokines that were considered not detected in our samples. For each condition, the median MFI and the range are reported. For each cytokine, the limit of detection (LOD) was computed as the MFI of the lowest standard -2* Standard Deviations of the lowest standard. Only IL-25 in Caco-2 cells stimulated with IFNγ had values above the LOD.

**Condition**	**Time (h)**	**IL-6 (LOD: 7.34)**	**IL-25 (LOD: 3.09)**	**IL-33 (LOD: 55.65)**	**TSLP (LOD: 10.54)**
**Resting**	6	0.25 [-1.00, 0.75]	-0.125 [-1.00, 0.50]	-1.25 [-2.00, -1.00]	0.75 [0.50, 1.50]
	24	0.25 [-0.75, 1.25]	-1.00 [-2.00, 0]	-1.00 [-2.00, -0.50]	1.00 [-1.50, 1.50]
	72	0.25 [-0.75, 1.25]	-0.50 [-1.00, 0]	-1.5. [-2.00, 0.50]	0 [-0.50, 0.50]
**Caco-2 + EVs**	6	-0.25 [-0.75, 0.25]	-1.25 [-3.00, 0]	-1.25 [-2.00, 0]	-0.50 [-1.00, 0]
	24	0 [-0.75, 0.25]	0 [0, 0]	-1.00 [-1.50, -1.00]	0 [-0.50, 0.50]
	72	0.25 [-0.75, 1.25]	-0.25 [-1.00, 1.00]	-1.00 [-1.00, -0.50]	0.50 [-0.50, 1.50]
**Caco-2 + iL3**	6	0.25 [-1.75, 0.75]	-0.50 [-1.00, 1.00]	-1.75 [-2.00, -1.00]	0 [-1.50, 0.50]
	24	0.25 [0.25, 2.75]	0 [-1.00, 0]	-2.00 [-2.00, -1.00]	0 [-1.50, 1.50]
	72	1.25 [0.75, 2.25]	0.50 [0, 2.00]	-1.25 [-2.00, -1.00]	0.25 [-0.50, 0.50]
**Caco-2 + IFNγ**	6	6.75 [2.25, 8.25]	**8.00 [3.50, 8.00]**	3.00 [1.00, 5.00]	3.50 [3.50, 4.50]
	24	5.25 [2.25, 6.25]	**6.00 [5.00, 8.00]**	2.00 [0, 5.00]	3.50 [3.50, 9.50]
	72	1.25 [0.75, 1.25]	**5.00 [4.00, 7.00]**	1.50 [1.00, 1.50]	2.00 [1.50, 2.50]
**iL3 only***	6	-0.75 [-0.75, -0.75]	-1.00 [-2.00, 0]	-2.00 [-2.00, -2.00]	0 [-0.50, 0.50]
	24	-0.25 [-0.75, 0.25]	-0.25 [-1.50, 1.00]	0 [-1.00, 1.00]	-0.50 [-0.50, -0.50]
	72	0.25 [-0.75, 1.25]	0.50 [0, 1.00]	-1.50 [-2.00, -1.00]	1.50 [0.50, 2.50]

Values in the table represent the median MFI and the range. Values above the LOD are reported in bold.
**iL3 only= il3 maintained without Caco-2 cells*

Figure S1. Representative images of *S. stercoralis* iL3 isolation from a clinical sample. A) Positive agar plate after 6 days of culture. Stools were mixed with charcoal and seeded on a *Strongyloides stercoralis* agar plate (Biolife). The tracks indicative of larval migration on the agar are clearly visible. B) Images of iL3 on the agar plate visualised at the stereomicroscope. C-E) Images of larvae in co-culture with Caco-2 cells visualised with Cytation 7 Imaging reader (BioTek) at 4X (C), 10X (D), 20X (E) magnification. Scale bar: 200µm. Red arrows in (C) indicate iL3.

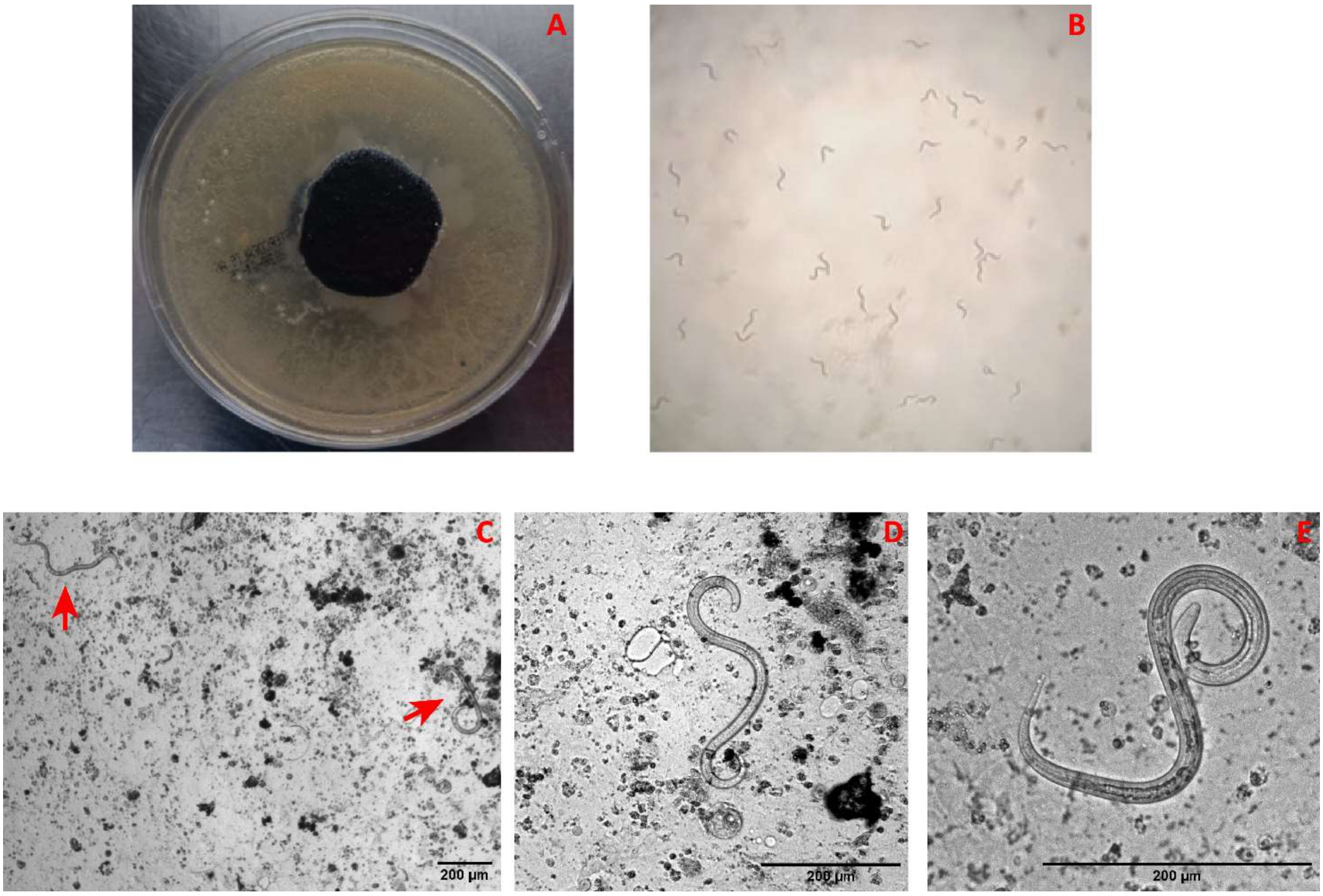

Figure S2. Volcano plot of transcriptomics results. Each plot represents the mRNA expression relative to resting conditions. Down-regulated transcripts (blue dots): FC≤2; p-value<0.05; Up-regulated transcripts (red dots): FC≥2; p-value<0.05; Gray dots: transcripts not regulated.

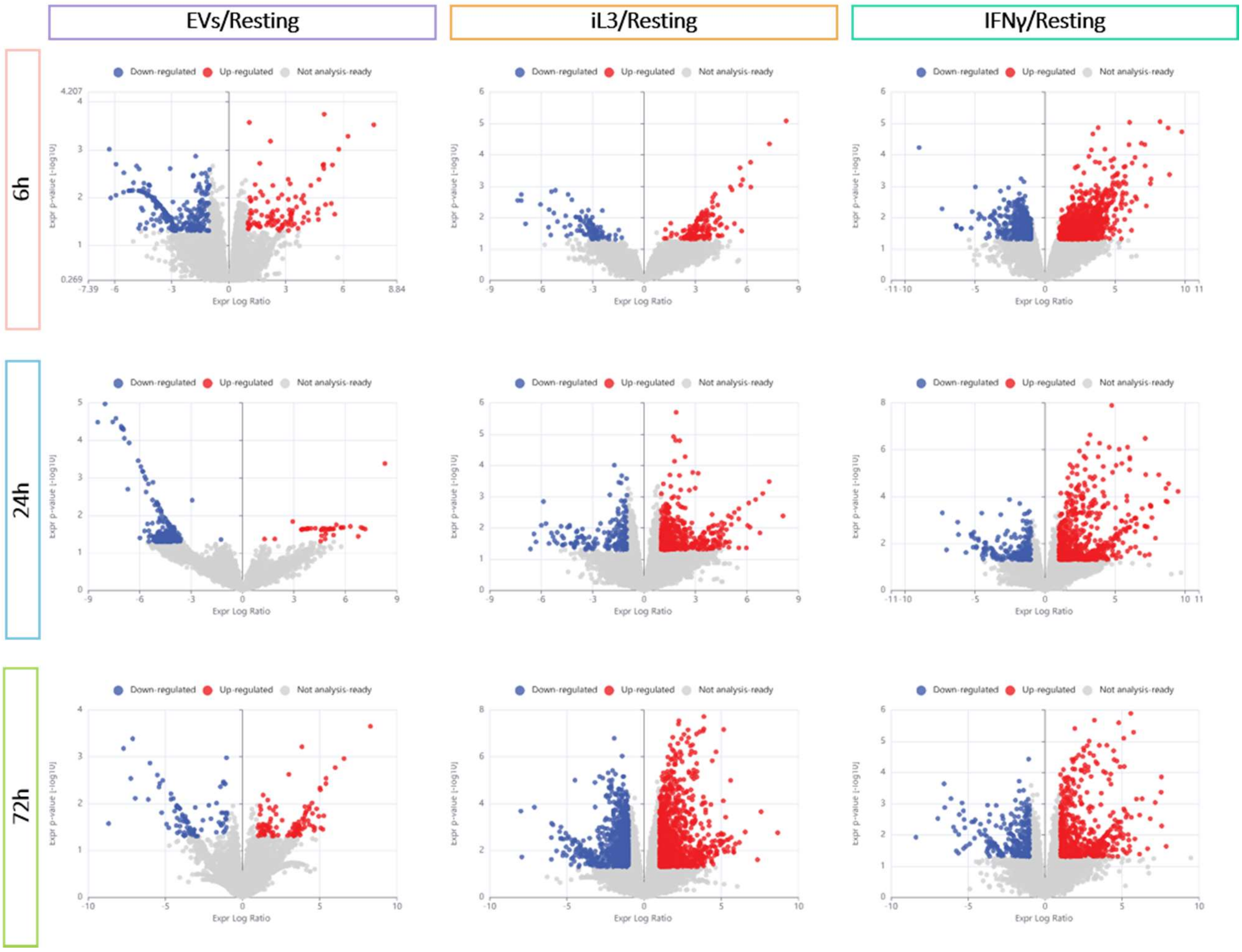

Figure S3. Volcano plot of proteomics results. Each plot represents the protein expression relative to resting conditions. Down-regulated proteins (blue dots): FC≤2; p-value<0.05; Up-regulated proteins (red dots): FC≥2; p-value<0.05; Gray dots: proteins not regulated.

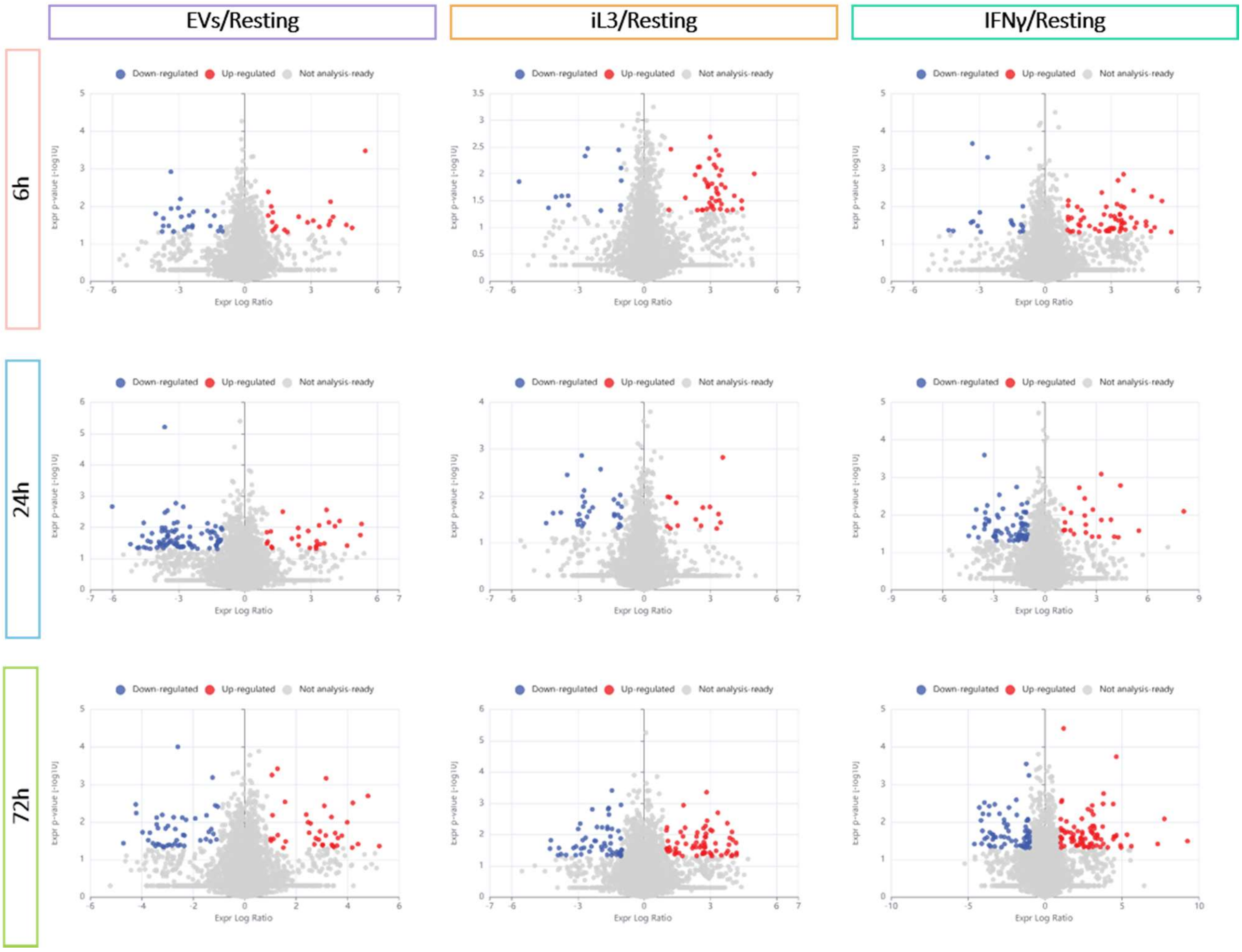

Figure S4. Cell viability following co-incubation of Caco-2 cells with either iL3, EVs or IFNγ. % viability of resting (i.e., untreated cells). At 6h and 24h both iL3 and EVs induced cell proliferation, while iL3 induced a slight reduction in cell viability 72h post exposure. Mean and SEM of 4 technical replicates.

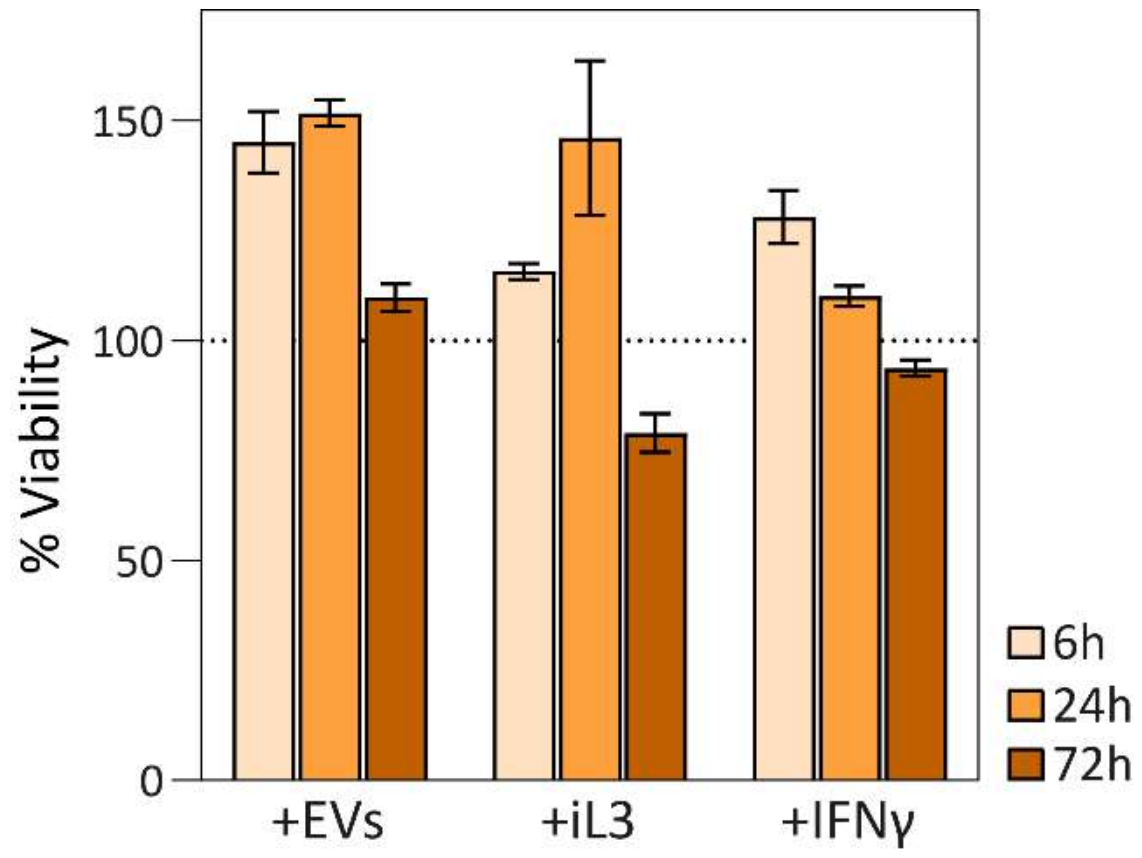

Figure S5. Top-5 pathways enriched in cells treated with IFNγ for 6, 24, or 72h. Enriched pathways were defined as having |Z-score| ≥2 and p-value <0.05. A) Top-5 pathways enriched among mRNA differentially expressed in IFNγ-treated cells compared to resting cells. B) Top-5 pathways enriched among proteins differentially expressed in IFNγ-treated cells compared to resting cells

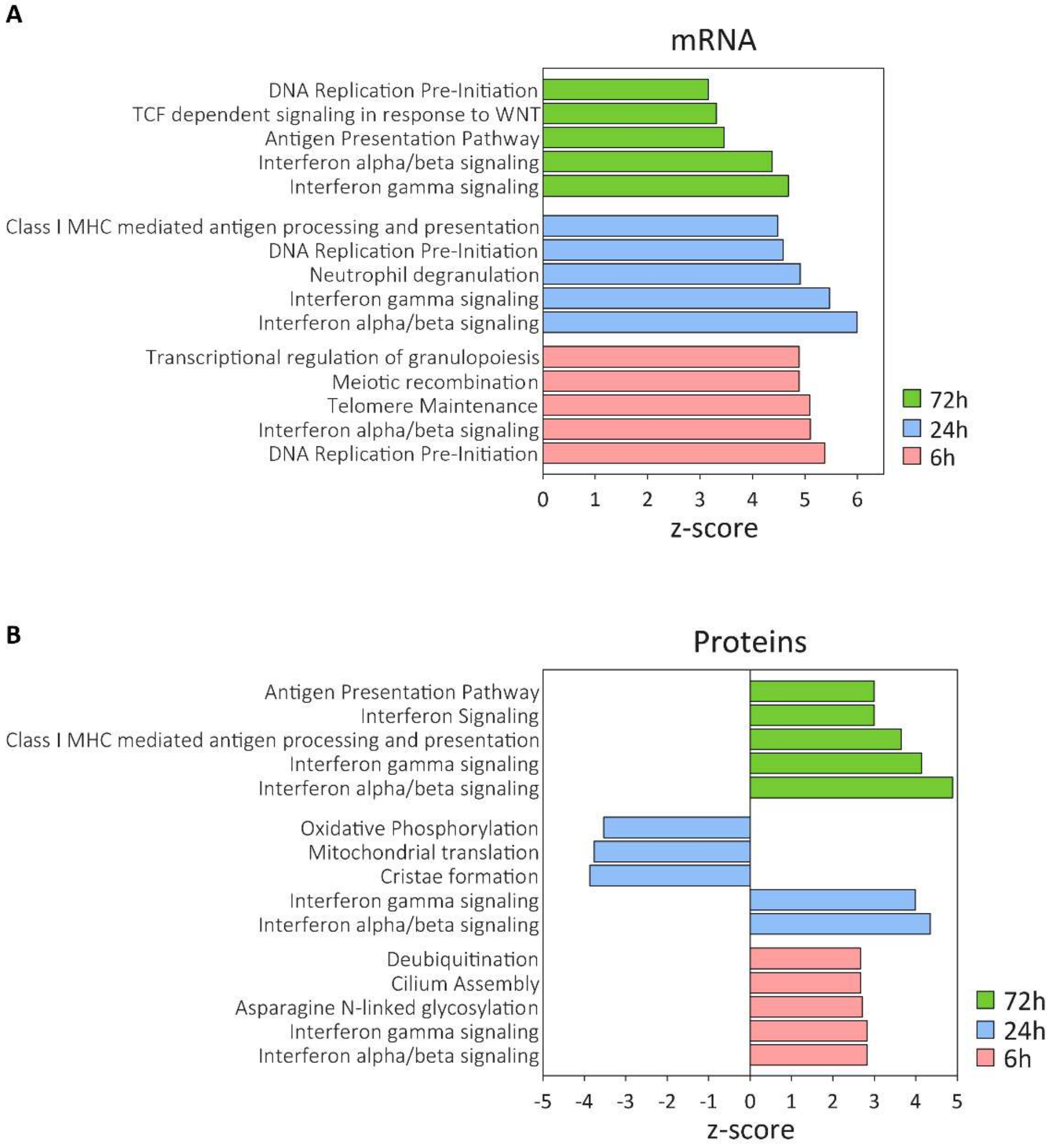

Supplement: Supplementary file 1 [file DataSheet3.pdf]
